# Developmental program‐independent secretory granule degradation in larval salivary gland cells of *Drosophila*

**DOI:** 10.1111/tra.12871

**Published:** 2022-11-29

**Authors:** Tamás Csizmadia, Anna Dósa, Erika Farkas, Belián Valentin Csikos, Eszter Adél Kriska, Gábor Juhász, Péter Lőw

**Affiliations:** ^1^ Department of Anatomy, Cell and Developmental Biology Eötvös Loránd University Budapest Hungary; ^2^ Institute of Genetics, Biological Research Centre Eötvös Loránd Research Network Szeged Hungary

**Keywords:** crinophagy, GARP, glue granule, lysosome, retrograde transport, Syx16, TGN

## Abstract

Both constitutive and regulated secretion require cell organelles that are able to store and release the secretory cargo. During development, the larval salivary gland of *Drosophila* initially produces high amount of glue‐containing small immature secretory granules, which then fuse with each other and reach their normal 3–3.5 μm in size. Following the burst of secretion, obsolete glue granules directly fuse with late endosomes or lysosomes by a process called crinophagy, which leads to fast degradation and recycling of the secretory cargo. However, hindering of endosome‐to‐TGN retrograde transport in these cells causes abnormally small glue granules which are not able to fuse with each other. Here, we show that loss of function of the SNARE genes *Syntaxin 16* (*Syx16*) and *Synaptobrevin* (*Syb*), the small GTPase *Rab6* and the GARP tethering complex members *Vps53* and *Scattered* (*Vps54*) all involved in retrograde transport cause intense early degradation of immature glue granules via crinophagy independently of the developmental program. Moreover, silencing of these genes also provokes secretory failure and accelerated crinophagy during larval development. Our results provide a better understanding of the relations among secretion, secretory granule maturation and degradation and paves the way for further investigation of these connections in other metazoans.

## Graphical abstract

Developmentally programmed crinophagy is necessary to eliminate obsolete glue‐containing secretory granules from the late larval salivary gland cells in *Drosophila*. Here, we show that, independently from the developmental program, crinophagy also acts in earlier larval stages, and removes immature secretory granules preventing their exocytosis in endosome‐to‐TGN retrograde transport‐deficient salivary gland cells. Abbreviations: EE–Early endosome, iSg–immature Secretory granule, Sg–Secretory granule.

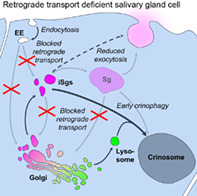

## 1 INTRODUCTION

Gland cells are specialized for high‐level production and release of several types of secretory materials such as hormones (by endocrine and neuroendocrine cells) or mucus and digestive enzymes (by exocrine cells) through the secretory pathway.^1^, ^2^, ^3^ Secretory granule formation and appropriate maturation are essential for storage and release of the produced secretory cargo. Additionally, secretory granules should grow to an adequate size for the successful and effective release of their content from the cell.

Gland cells function in cycles during which accumulation and, in response to external signals, a subsequent discharge of secretory granules happen in them. Secretory proteins are initially concentrated in vesicles derived from the enlarged Golgi complex and stored in a homogeneous population of spherical secretory granules that fuse with each other before release.^4^ Finally, these secretory granules become competent to fuse with the plasma membrane and, as a result, they release their cargo into the extracellular space.

In addition to hormone and enzyme production, secretion of mucus is also essential for the organism. Mucopolysaccharide‐containing large secretory granules are formed at high levels by several types of gland cells and organs at certain locations in the animal and human body. Important examples are mucus production to protect the wall of the stomach or to advance feces in the colon.^5^, ^6^, ^7^ Insufficient mucus secretion causes disorders like gastric ulcer or constipation. Importantly, the homeostatic equilibrium of secretory tissues and cells requires not only formation, maturation and secretion but also degradation of secretory granules and materials. Understanding these processes is essential to avoid the gland cell and organ‐connected diseases, such as diabetes and acute pancreatitis.^8^, ^9^

The larval salivary gland of *Drosophila melanogaster* is an excellent and frequently used model system for the secretory granule biology research.^10^, ^11^, ^12^ During larval development, some of these gland cells produce and secrete so‐called glue proteins required for attaching the forming pupa to a solid surface.^10^, ^13^, ^14^ The tiny presecretory granules originate from the trans‐Golgi network (TGN) and then their homotypic fusion is activated.^11^, ^15^ As a result, glue proteins are stored in very large vesicles preceding the secretion, ranging in size from 3 to 3.5 μm in diameter.

The molecular background of secretory granule formation, maturation and homotypic fusion is a highly and precisely regulated mechanism which is better and better understood.^16^ Components of vesicular trafficking pathways, such as vesicle coat and adaptor proteins,^17^, ^18^ small GTPases,^19^, ^20^ molecular motors,^21^ tethering complexes^22^ and SNARE (Soluble NSF Attachment Protein REceptor) proteins^23^ are necessary for these processes. In the salivary gland cells of *Drosophila* larvae, the early stage of secretory granule formation is strongly connected to the TGN and requires tight cooperation of the vesicle coat protein clathrin and its adaptor protein complex AP‐1.^11^ Moreover, playing an important role in recruiting the members of the AP‐1 complex, the small GTPase, Arl1 is also necessary for this step.^24^ In addition, the early endosomal compartment and the endosome‐to‐TGN retrograde transport mechanisms strongly contribute to the formation and maturation of secretory granules, too.^25^, ^26^ The trans‐Golgi‐related Qa‐type SNARE protein (Q‐SNAREs that have a glutamine in the zero ionic layer of the assembled complex), Syntaxin 16 (Syx16) and the members of the GARP (Golgi‐Associated Retrograde transport Protein) tethering complex (Vps51, Vps52, Vps53 and Vps54) all have a role in these latter processes.^25^, ^27^, ^28^, ^29^, ^30^, ^31^ The function of Rab1 and Rab11 small GTPases as well as the lipid kinase PI4KIIα in the maturation and granule‐to‐granule fusion of glue‐containing secretory granules has also been confirmed in *Drosophila* larval salivary gland cells.^32^, ^33^

Production and release of the mucus type of glue in larval salivary gland cells are triggered by an ecdysone hormone pulse which, in turn, is regulated by the activity of the transcriptional repressor Bab2 during postembryonic development.^10^, ^34^ Interestingly, fusion with the apical cell membrane and subsequent release of glue into the gland lumen require vesicle contraction, and the necessary force is mediated by an actin coat formation and recruitment of myosin around each vesicle.^35^, ^36^ Importantly, earlier studies clearly showed that components of the endosome‐to‐TGN retrograde transport, such as Vps52, Vps53 and Rab6 are also needed for normal secretory process.^37^, ^38^, ^39^

Degradation of unnecessary, obsolete, or damaged secretory granules is required for maintaining homeostatic equilibrium in gland cells and tissues. There are several possibilities for their elimination, depending mainly on granule size.^2^, ^40^, ^41^ Macroautophagy is the best known autophagic process during which a double membrane cistern (also known as phagophore) creates a double membrane bounded vesicle (an autophagosome) which incorporates parts of the cytoplasm and then able to fuse with lysosomes to degrade its content.^42^, ^43^ In gland cells, autophagosomes was shown also include small secretory granules, such as insulin‐containing β‐granules in case of pancreatic β‐cells.^2^, ^8^, ^40^ In contrast, microautophagy involves invagination of the lysosomal membrane into the lumen, engulfing the surrounding cytoplasm and creating small intraluminal vesicles with cytoplasmic content.^44^, ^45^ These intraluminal vesicles can also contain small secretory granules, for example, insulin‐containing β‐granules in pancreatic cells, which subsequently are degraded in the lumen of the lysosome.^2^, ^8^, ^40^ Nevertheless, the main autophagic process used by gland cells for elimination of obsolete or damaged secretory granules is crinophagy, during which granules directly fuse with late endosomes or lysosomes.^2^, ^8^, ^12^, ^40^, ^46^, ^47^ Moreover, the failure of secretion causes abnormally intensive secretory granule degradation, which mainly occurs via crinophagy.^8^, ^48^ Importantly, crinophagy creates a special type of secondary lysosome known as crinosome, with low pH, active lysosomal hydrolases and degrading secretory content.^40^, ^49^

Crinophagy was discovered 56 years ago by electron microscopic examination of anterior pituitary gland cells in rats.^46^ This tightly regulated catabolic process is part of the normal physiology and development of exocrine, endocrine and neuroendocrine cells to control the secretory granule pool.^2^ The late larval salivary gland of *Drosophila* is an excellent model to study underlying molecular mechanisms. After the burst of secretion of glue proteins, the unnecessary glue‐containing secretory granules remained in the cytoplasm are degraded via crinophagy. Normally, this process is developmentally regulated and may contribute to recycling of the residual secretory cargo, preceding full disruption of the larval salivary gland that occurs before metamorphosis.^12^, ^50^ The first molecules, involved in vesicular trafficking and vesicle fusion (small GTPases, tethering complex members, SNARE proteins), which control glue granule‐lysosome fusion in the late larval salivary gland cells of *Drosophila melanogaster* were identified earlier by us. We have discovered the role of the HOPS (HOmotypic fusion and Protein Sorting) tethering complex, Rab7 and Arl8 small GTPases in this process. Moreover, we have also identified a new SNARE complex containing Syntaxin 13 (Syx13), Snap29 and Vamp7—as Qa, Qbc and R type of SNARE proteins, respectively, which are all required in the developmentally regulated glue granule‐lysosome fusion in salivary gland cells of *Drosophila*.^12^, ^51^ It is important to note that, the unnecessary or obsolete proinsulin containing secretory vesicles may also fuse with lysosomes for fast degradation of the secretory cargo in pancreatic β cells. This process is mediated by a recently identified other type of SNARE complex containing Syx7 (Qa), Vti1B (Qb), Syx8 (Qc) and Vamp4 (R) SNARE proteins. The difference between the compositions of the SNARE complexes of glue granule and β granule crinophagic degradation reflects to the high diversity of the secretory cells and vesicles considering the secretory cargo.^52^ Interestingly, the molecular signal, which directs unnecessary or obsolete glue granules for crinophagic degradation instead of their exocytosis is remained unknown.

In this study, we show that the failure of endosome‐to‐TGN retrograde trafficking involved in recycling of membrane proteins and lipids among the endosomal, secretory and TGN compartments, not only causes defects in secretory granule formation, maturation and secretion but also activates early crinophagic degradation of these immature glue granule forms. These observations strongly contribute to better understanding of the molecular background of targeting unnecessary or damaged secretory granules for crinophagic degradation and may help to find an answer how this process is activated in the larval salivary gland cells of *Drosophila*.

## 2 RESULTS

### 2.1 Glue granule formation, maturation and degradation are developmentally programmed in *Drosophila*

The biogenesis, development and breakdown of the large mucopolysaccharide‐containing secretory granules are complex, multistep mechanisms. We used an earlier developed “GlueFlux” system for the examination of the developmentally programmed glue granule acidification and breakdown in the late larval salivary gland cells of *Drosophila*.^12^, ^40^ Salivary gland cells of our transgenic *Drosophila* stock co‐express Glue‐GFP and Glue‐DsRed recombinant proteins labeling secretory cargo‐containing granules (the special glue protein used in the system is Sgs3, Salivary gland secretion 3).^10^, ^12^, ^53^ Following the fusion of recombinant glue‐containing granules with late endosomes or lysosomes and formation of crinosomes, the GFP fluorescence is quenched in the acidic environment. In contrast, the Glue‐DsRed component is less sensitive to the low pH, thus the degrading glue content retain the DsRed signal. Using this system, we can distinguish the intact (GFP‐ and DsRed‐positive) granules from crinosomes (DsRed signal only).^12^, ^40^

**FIGURE 1:**
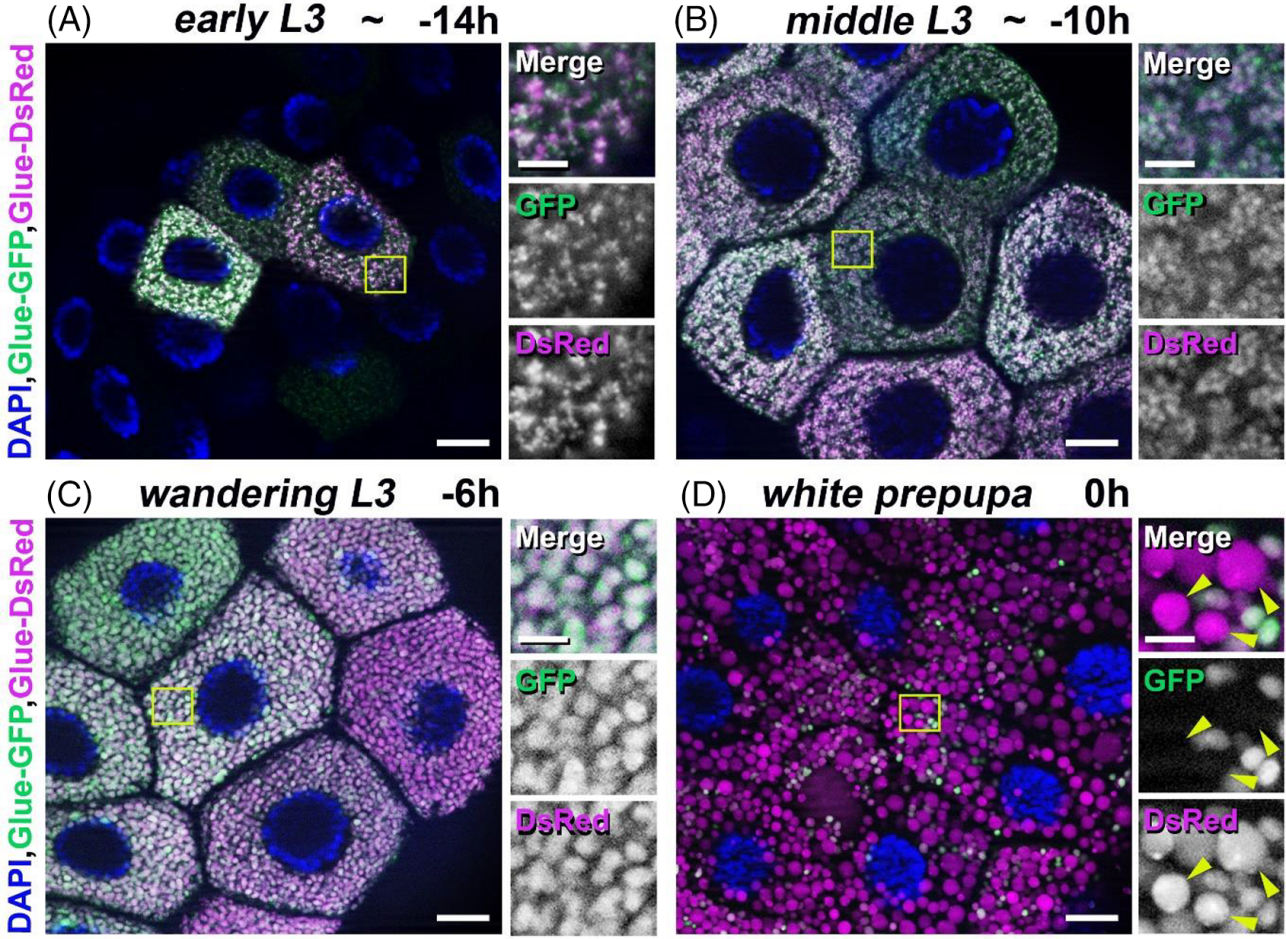
Time course of developmentally programmed formation, maturation and degradation of glue‐containing secretory granules in *Drosophila* salivary gland cells. (A‐D) From birth to death of glue granules in salivary gland cells co‐expressing the Glue‐GFP and Glue‐DsRed (GlueFlux) reporters. (A) Early L3 stage (~ −14 h RPF) salivary gland cells contain very small (0.1–0.5 μm), intact (both GFP‐ and DsRed‐positive) pre‐secretory vesicles (primordia). (B) Mid‐L3 stage (~ −10 h RPF) salivary gland cells are filled with larger (0.5–1 μm), intact, clustered, immature glue granules. (C) Wandering L3 stage (−6 h RPF) salivary gland cells contain even larger (3–3.5 μm) glue granules still positive for Glue‐GFP and Glue‐DsRed. (D) In the salivary gland cells from white prepupa (0 h RPF) stage animals, there are huge (4–8 μm) glue granules (crinosomes), mostly with a degrading content positive for DsRed only indicated by yellow arrowheads in the right insets. The boxed regions in panels A‐D are shown enlarged on the right side of each panel. Green and magenta channels of merged images are also shown separately as indicated. Bars: 20 μm (A–D), 5 μm (A–D right insets)

We defined the larval developmental stages of salivary glands of *Drosophila melanogaster* as early L3 developmental stage (approximately at ‐14 h RPF [relative to puparium formation], primordia of glue granules appear in at least 1–2 salivary gland cells; however, there are no granules in no less than 1–2 cells), middle L3 developmental stage (approximately at ‐10 h RPF, small granules are present in all salivary gland cells), wandering L3 stage (approximately at ‐6 h RPF, fully mature granules) and white prepupal stage (0 h RPF, most of the glue granules are degraded). Larval salivary glands begin to produce high amount of immature form of glue granules at early L3 developmental stage. We observed that these granule primordia are very small (0.1–0.5 μm), localized near the TGN and must be intact, because they are positive for Glue‐GFP and Glue‐DsRed (Figure 1A). Later, at middle L3 developmental stage, these granules become larger (0.5–1 μm) presumably because of the homotypic fusion of granule primordia. Importantly, these small immature granules are still GFP‐ and DsRed‐positive, thus intact (Figure 1B). At the so‐called wandering larval stage, the intact glue granules reach their final size (3–3.5 μm) as a result of further homotypic fusions and aggregation events. These mature forms are ready for exocytosis (Figure 1C). After the peak of glue secretion at white prepupal stage, the unnecessary or obsolete secretory granules remained in the cytoplasm must become acidified, because most of the detected glue containing structures were positive only for DsRed. These granules were measured to be larger (5–8 μm) than the intact (both GFP‐ and DsRed‐positive) ones (Figure 1D).

These observations show that glue granule formation, maturation and degradation are developmentally programmed, consecutive mechanisms in *Drosophila* salivary gland cells.

### 2.2 Proteins involved in the retrograde transport from endosome‐to‐TGN are required for glue granule maturation and prevent early granule acidification and glue degradation

Contribution of the endocytic compartment and functional retrograde transport from endosome‐to‐TGN is necessary for normal secretory granule maturation and exocytotic process. Based on this fact, we hypothesized that—independently from the developmental program—abnormal secretory granule formation or failure in secretion may induce premature secretory granule acidification and glue degradation in retrograde transport‐deficient salivary gland cells of wandering (−6 h RPF) animals. Therefore, we examined consequences of the loss of function of some endosome‐to‐TGN retrograde transport components. These proteins include SNAREs, tether complex members and small GTPases involved in membrane fusion processes.

**FIGURE 2:**
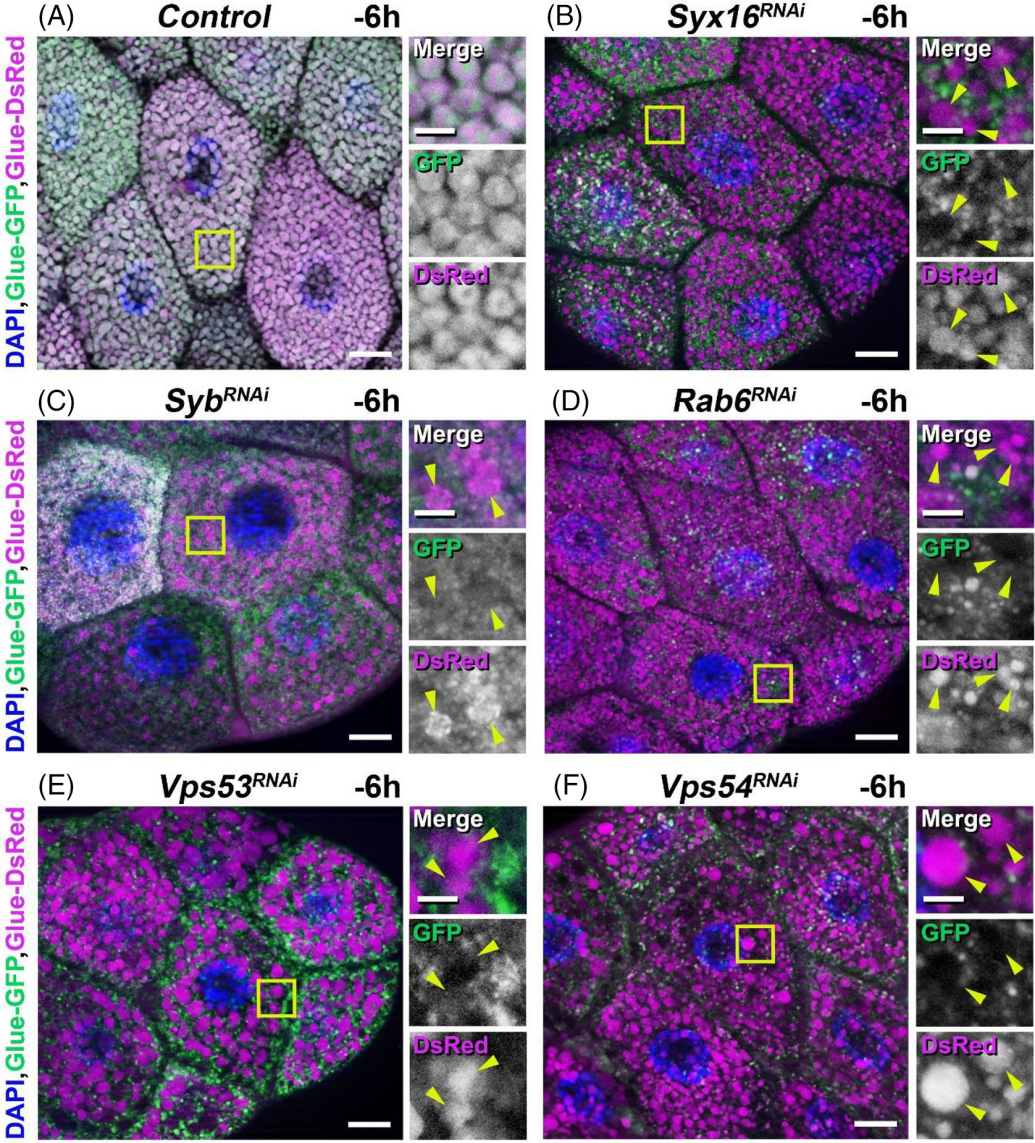
Loss of function of genes involved in the endosome‐to‐TGN retrograde transport and secretory granule maturation leads to early acidification and breakdown of immature glue granules in *Drosophila* salivary gland cells at the wandering larval stage of development. (A–F) Degradation of very small glue granules in the salivary gland cells of wandering L3 (−6 h RPF) animals co‐expressing Glue‐GFP/Glue‐DsRed (GlueFlux) reporters together with an RNAi construct for one of the genes involved in retrograde transport‐ and secretory granule maturation. (A) Control wandering L3 stage (−6 h RPF) larval salivary gland cells normally enclose large (3–3.5 μm), intact (GFP‐ and DsRed‐ double positive) glue‐containing secretory granules. Compared to the control, salivary gland cells containing the RNAi construct for the SNARE‐proteins *Syx16* (B), and *Syb* (C), the small GTPase *Rab6* (D), and key members of the GARP tethering complex *Vps53* (E) and *Vps54* (F) contain very small (0.1–1 μm), immature, intact secretory vesicles which are positive for both GFP and DsRed. Interestingly, these cells also include 4–8 μm acidic (positive for DsRed only) degradative glue granules indicated by yellow arrowheads in the right insets. The boxed regions in panels A‐F are shown enlarged on the right side of each panel. Green and magenta channels of merged images are also shown separately as indicated. Bars: 20 μm (A–F), 5 μm (A–F right insets)

Firstly, using the “GlueFlux” system, we tested the result of silencing of the trans‐Golgi‐related Qa SNARE Syx16 in salivary gland cells at the wandering larval stage of development (−6 h RPF). These cells normally contain large and intact glue granules 3–3.5 μm in size, positive for both GFP and DsRed signals and ready for secretion (Figures 1C and 2A). As expected, loss of function of Syx16 inhibits homotypic fusion and causes abnormally, reduced size (<0.1–1 μm) and Glue‐GFP/Glue‐DsRed‐positive (thus intact) glue granule primordia. Interestingly, these silenced cells are also filled with high amount of large (3–6 μm) granules positive only for Glue‐DsRed, indicating that these glue containing structures are acidic and their content is under degradation (Figure 2B).

We also investigated another SNARE protein, the R‐SNARE (R‐SNAREs that have an arginine in the zero ionic layer of the assembled complex), Synaptobrevin (Syb) which (as an orthologue of the mammalian Vamp1, Vamp2 and Vamp4) has a function in exocytosis as well as in retrograde transport and the small GTPase Rab6, which also has a role in retrograde trafficking.^30^, ^54^ Silencing of Syb and Rab6 showed similar phenotypes to the effect of Syx16 RNAi: compared with the control, we detected small (0.1–1 μm), immature GFP‐ and DsRed‐positive glue granule primordia and abnormally early appeared (−6 h RPF instead of 0 h RPF) acidic (only DsRed‐positive) larger (2–5 μm) crinosome‐like structures in the cytoplasm of the silenced salivary gland cells (Figure 2C, D).

Moreover, silencing two key members of the GARP tethering complex, Vps53 and Vps54 (Scattered in *Drosophila*) also causes accumulation of intact (GFP‐ and DsRed‐positive) small (0.1–0.5 μm) glue granule primordia and large (5–6 μm), only DsRed‐positive crinosome‐like structures in the gland cells (Figure 2E, F).

Using our “GlueFlux” system, we also tested some earlier identified factors^11^, ^24^, ^33^ of glue granule maturation, such as the small GTPase Arl1, the clathrin adaptor complex subunit AP‐1γ, the clathrin heavy chain component Chc and the lipid kinase PI4kIIα. We found that—similarly to our previous results—silencing of these genes caused strong glue granule maturation defect and early, fervent acidification and degradation of glue in the gland cells (Figure S1A–E).

Interestingly, increased Ca^2+^ concentration is required for several types of vesicular trafficking, such as exocytosis and clathrin mediated endocytosis (CME). It is also known that, an endocytic contribution is necessary for the homotypic fusion of glue granules during secretory granule maturation.^25^ Thus, we hypothesized that if a Ca^2+^ surge is needed for CME, it is possibly required for secretory granule homotypic fusion by providing normal CME as well, thus silencing of the relevant Ca^2+^ channel gene may also perturb secretory granule maturation. We have identified the role of Flower (Fwe) in the regulation of glue granule maturation as a protein that, in a multimer form, creates a Ca^2+^ channel. Silencing of the Fwe gene also caused similar accumulation of immature small, intact glue granules and early appearance of acidic crinosome‐like structures in the salivary gland cells at the wandering stage of development (Figure S1F).

These results indicate that in the salivary gland cells of *Drosophila*, failure of the endosome‐to‐TGN retrograde trafficking causes strong defects in secretory granule formation. In addition, these abnormally accumulated, immature granules are prematurely diverted to the acidification and degradation pathway independently from the developmental program.

### 2.3 In the absence of the endosome‐to‐TGN retrograde traffic, immature and small glue granules are degraded via crinophagy in *Drosophila* salivary gland cells

Elimination of unnecessary or damaged secretory granules in gland cells involves mainly autophagic mechanisms, such as macro‐ and microautophagy as well as crinophagy. We examined these three main degradation possibilities to explore how immature secretory granules (primordia) could be acidified and degraded in the endosome‐to‐TGN retrograde transport‐deficient *Drosophila* salivary gland cells. To investigate this, we used an earlier developed fluorescent system which is suitable to follow secretory granule‐lysosome fusion.^12^, ^40^ This dual construct contains the previously described Glue‐DsRed to label secretory granules and a GFP‐tagged Lysosome Associated Membrane Protein‐1 (GFP‐Lamp1), as a late endosomal/lysosomal marker. Fusion of a secretory granule and a late endosome/lysosome appears as a GFP‐positive ring‐like structure (representing the late endosomal/lysosomal membrane) around a DsRed‐positive spot (glue material) which is the sign of crinosome formation.^12^, ^40^ It is important to note that, the large crinosome‐like structures are not positive for GFP‐Lamp1, because in this construct, the GFP part faced to the luminal side of lysosomes/crinosomes and was rapidly quenched in the acidic, degradative milieu.^12^, ^55^

**FIGURE 3:**
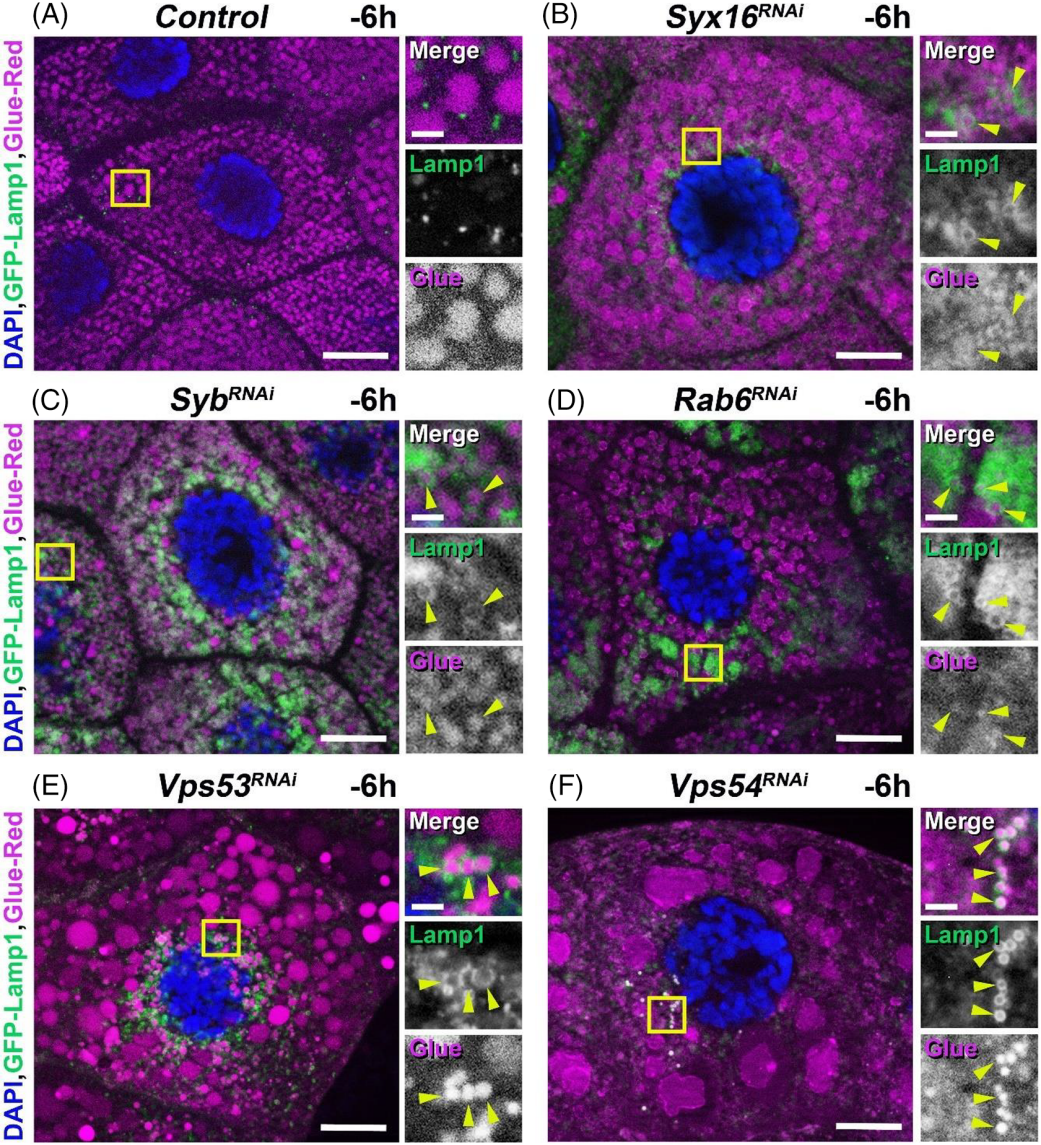
Developmental program‐independent degradation of immature glue granules take place via crinophagy. (A–F) Premature degradation of very small glue granules in the salivary gland cells of wandering L3 (−6 h RPF) animals co‐expressing Glue‐DsRed and GFP‐Lamp1 reporters together with an RNAi construct for one of the genes involved in retrograde transport and secretory granule maturation. GFP‐Lamp1‐positive ring formation around the Glue‐DsRed‐containing secretory vesicles is the hallmark of crinophagic activity. (A) Control wandering L3 stage (−6 h RPF) larval salivary gland cells normally contain large (3–3.5 μm) glue granules and very few late endosome‐ or lysosome‐like GFP‐positive dots. Compared to control, salivary gland cells containing the RNAi construct for the SNARE protein, *Syx16* (B), and *Syb* (C), the small GTPase, *Rab6* (D) and the key members of the GARP tethering complex (*Vps53* – E and *Vps54* – F) comprise GFP‐Lamp1‐positive ring‐like structures around the very small Glue‐DsRed‐positive dots, which indicate the previously formed small crinosomes (showed by yellow arrowheads in the right insets) and the premature activity of crinophagy. Moreover, these cells also include larger, only Glue‐DsRed‐positive dots, presumably related to old crinosomes, because in these acidic‐degradative structures the GFP component of the Lamp1 fusion protein can be rapidly quenched. The boxed regions in panels A‐F are shown enlarged on the right side of each panel. Green and magenta channels of merged images are also shown separately as indicated. Bars: 20 μm (A–F), 3 μm (A–F right insets)

Control salivary gland cells contained normal‐sized glue granules with no detectable GFP‐Lamp1 ring around them (Figure 3A). Compared to control, gland cells with silenced Syx16 contained large (3–6 μm) Glue‐DsRed structures which should be acidic, based on our previous “GlueFlux” results and were also filled with abnormally small secretory vesicles (Figure 2B). Interestingly, some of the immature granules contained GFP‐Lamp1 in their membrane (Figure 3B). This phenomenon may indicate that these vesicles directly fuse with late endosomes/lysosomes which is the hallmark of crinophagy and suggests that this is the process that leads to the acidification and degradation of these granules.

Moreover, silencing of the other examined retrograde transport components, Syb, Rab6, Vps53 and Vps54 showed similar phenotype to Syx16 RNAi cells: large crinosome‐like structures and small glue granules were seen in the cytoplasm and some immature glue granules accumulated GFP‐Lamp1 in their membrane (Figure 3C–F, respectively).

These results strongly indicate that deficiencies in the retrograde transport mechanisms cause formation of abnormally small glue granules which are eventually degraded via crinophagy at the wandering developmental stage preceding (and thus independently from) the normal developmental program.

### 2.4 Ultrastructure of the endosome‐to‐TGN retrograde transport‐deficient salivary gland cells in *Drosophila*

**FIGURE 4:**
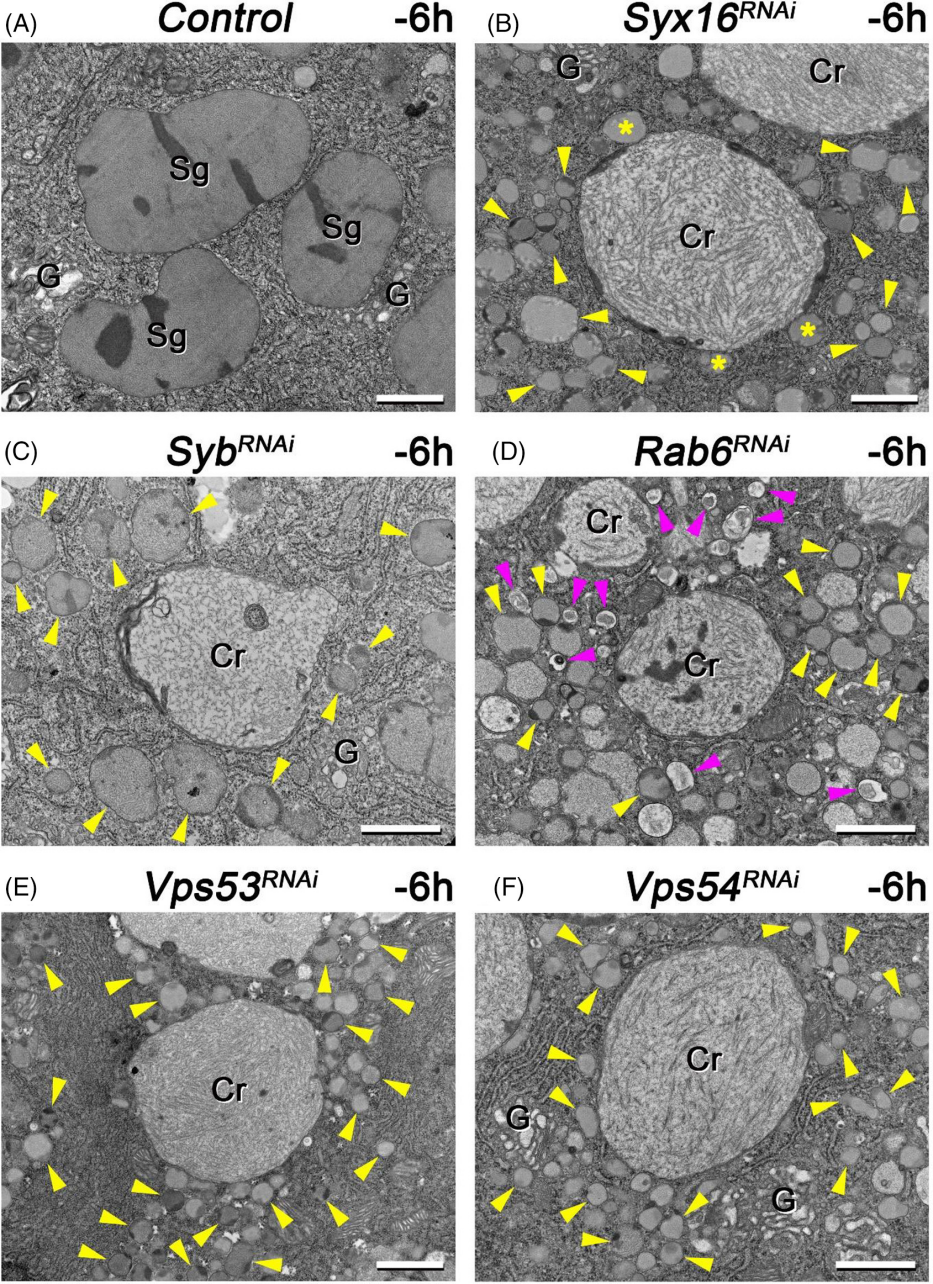
Ultrastructure of the endosome‐to‐TGN retrograde transport‐deficient salivary gland cells isolated from wandering *Drosophila* larvae. (A) Salivary gland cells from control wandering larvae include large (3–3.5 μm), spotty mucopolysaccharide (glue protein) containing, round secretory granules (Sg). Parts of the Golgi complex (G) are also seen in the cytoplasm. (B) Silencing of the *Syx16* gene causes appearance of abnormally small (0.1–1 μm), immature and intact glue granules (labeled by yellow arrowheads), together with large, electron‐lucent, crinosome‐like structures with loose, filamentous content (Cr). Yellow asterisks indicate fusion of immature glue granules with a crinosome. (C) Loss of function of *Syb* results in larger (0.5–1.5 μm), intact but immature (abnormally small), glue granules (yellow arrowheads) and crinosomes with diverse inner content. (D) Interestingly, *Rab6* RNAi cell is also filled with immature secretory granules (yellow arrowheads) and larger crinosomes. Moreover, these cells also contain high amount of autophagic structures in the cytoplasm (magenta arrowheads in panel D). Silencing of *Vps53* (E) and *Vps54* (F) also resulted in accumulation of small glue granules (primordia – indicated by yellow arrowheads) and large crinosomes with loose inner structure. Abbreviations: Cr – Crinosome, G – Golgi apparatus, Sg – Secretory granule. Bars: 1 μm (A–F)

For further analysis of retrograde transport failure consequences in larval salivary gland, we examined the ultrastructure of the different RNAi‐treated cells. In the cytoplasm of control cells, we detected large (3–3.5 μm), mature, spotty, round glue granules. Other signs of the high secretory activity were also present in these cells, such as extensive rough endoplasmic reticulum and some Golgi regions (Figure 4A).^1^

Compared to control, the Syx16‐silenced cells were filled with very small (0.1–1 μm), immature glue granules. Interestingly, the ultrastructural hallmark of intact glue granules is the spotty look^12^ which was also observable in case of the immature granules in Syx16 RNAi‐treated cells. Moreover, there were some larger, electron‐lucent structures with loose filamentous content (glue under digestion^12^)‐presumably crinosomes. Additionally, some of these structures contained electron‐dense material (dark spots) in their cortical region which might represent remnants of secretory granule content (Figure 4B).

Phenotype of Syb silencing showed similarity to that of Syx16 RNAi, although, most of the immature glue granules were a little bit larger (0.5–1.5 μm) than in Syx16 RNAi cells. Importantly, crinosome‐like structures were also present in these cells. Interestingly, one of the putative crinosomes contained an inclusion body which might originate from autophagosome‐crinosome fusion (Figure 4C).

Silencing of the small GTPase, Rab6 gene also produces crinosomes and immature, small (0.1–1 μm) and spotty glue granules in the cytoplasm. Besides this, autophagic structures, which often contain cytoplasmic material under degradation, also appeared in these cells (Figure 4D).

Finally, we have examined the absence of two of the key components of GARP tethering complex, Vps53 and Vps54. Loss of function of these genes resulted in very small (0.1–0.5 μm), primordial glue granule accumulation and premature crinophagic activity, which was indicated by the presence of the crinosomes with loose filamentous, electron‐lucent content (Figure 4E, F).

These ultrastructural analyses strongly support our model suggesting that failure in secretory granule maturation process (including homotypic fusion) causes premature crinophagy, activated independently of the normal developmental program. Supposedly, endosome‐to‐TGN retrograde transport‐deficient salivary gland cells use this mechanism to remove accumulated immature glue granules from their cytoplasm.

### 2.5 Examination of developmental program‐dependent and independent crinophagy in *Drosophila*

**FIGURE 5:**
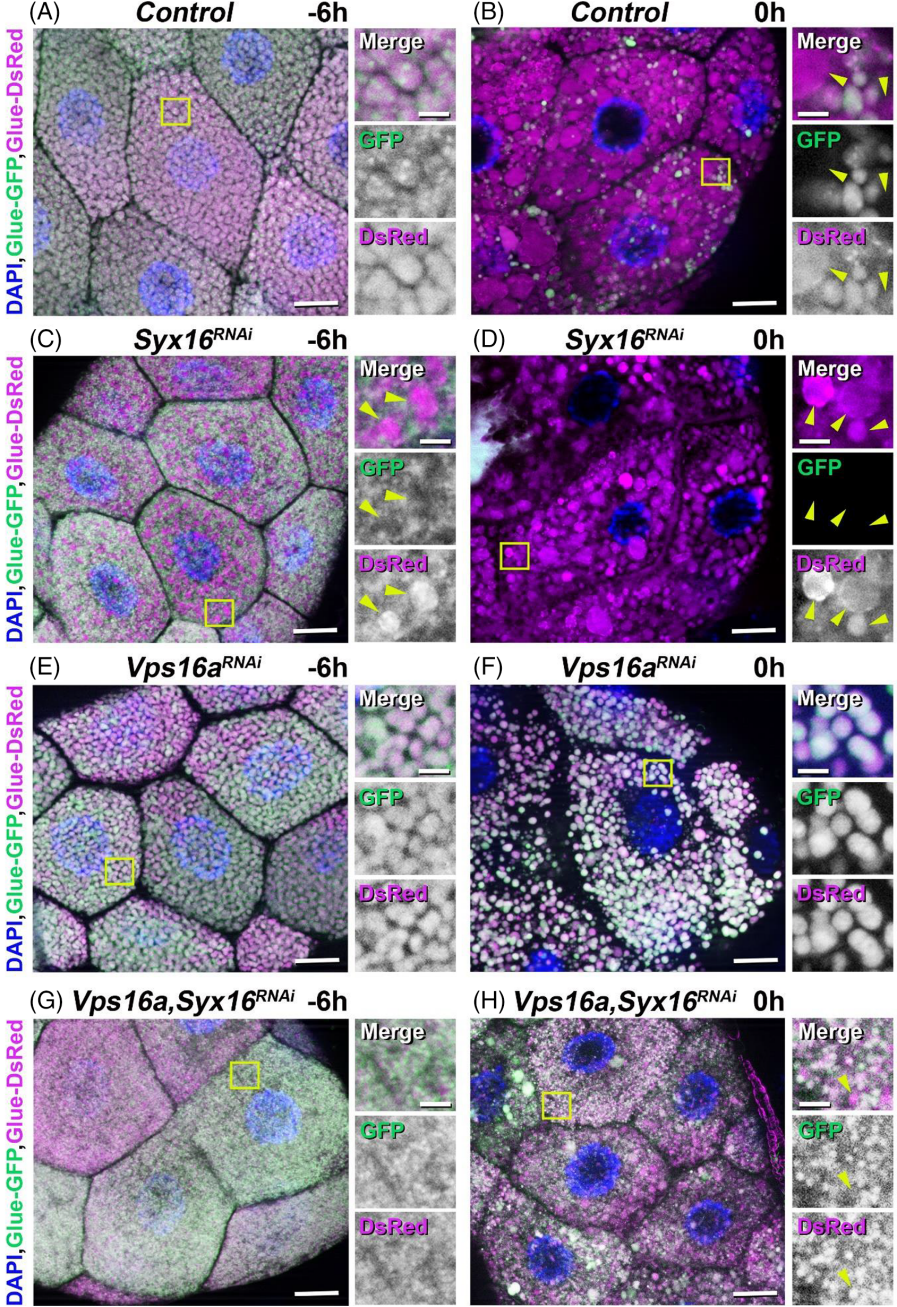
Effects of the developmental program‐dependent and ‐independent crinophagy in salivary gland cells deficient for endosome‐to‐TGN retrograde transport and glue granule‐lysosome fusion. (A–B) Developmentally programmed secretory granule acidification and degradation in *Drosophila* prepupal salivary gland compared to an earlier stage. (A) Control larval salivary gland cells are filled with large (3–3.5 μm) and intact (both GFP‐ and DsRed‐positive) glue granules at wandering stage (−6 h RPF). (B) During prepupa formation (0 h RPF), most of the glue granules become acidic (marked by the presence of only DsRed‐positive, huge, 4–8 μm structures) and get degraded via crinophagy in the control sample. (C–D) Accelerated crinophagy in *Syx16*‐silenced salivary gland cells. (C) Loss of *Syx16* function causes the accumulation of immature (0.1–1 μm), intact, glue‐containing granules and large (4–8 μm), acidic structures in these cells which suggests premature crinophagy at wandering (−6 h RPF) stage. (D) Developmentally programmed but abnormally accelerated glue granule degradation is present in *Syx16*‐silenced salivary gland cells of prepupal animals (0 h RPF). These cells contain acidic, large crinosome‐like structures exclusively, immature, intact glue granules cannot be detected at all. (E–F) Crinophagy is absent from *Vps16a*‐silenced salivary gland cells. (E) White normal‐sized secretory vesicles are formed even in *Vps16a* RNAi condition at the wandering stage of development, but the developmentally programmed degradation of these granules is damaged, because most of the glue granules remain positive for both GFP and DsRed at prepupal stage (F). (G–H) Salivary gland cells simultaneously silenced for *Vps16a* and *Syx16* lack the huge, only DsRed‐positive acidic structures present in only *Syx16* RNAi‐treated or 0 h RPF control cells. Under double RNAi conditions, cells were filled mostly with small, immature and intact glue granules both in wandering (G) and prepupal (H) animals. Importantly, these cells did not contain any large acidic (only DsRed‐positive) crinosome‐like structures at both of the two examined developmental stages. The acidic (positive for DsRed only) glue granules (crinosomes) are labeled by yellow arrowheads in the right insets. The boxed regions in panels A–H are shown enlarged on the right side of each panel. Green and magenta channels of merged images are also shown separately as indicated. Bars: 20 μm (A–H), 5 μm (A–H right insets)

**FIGURE 6:**
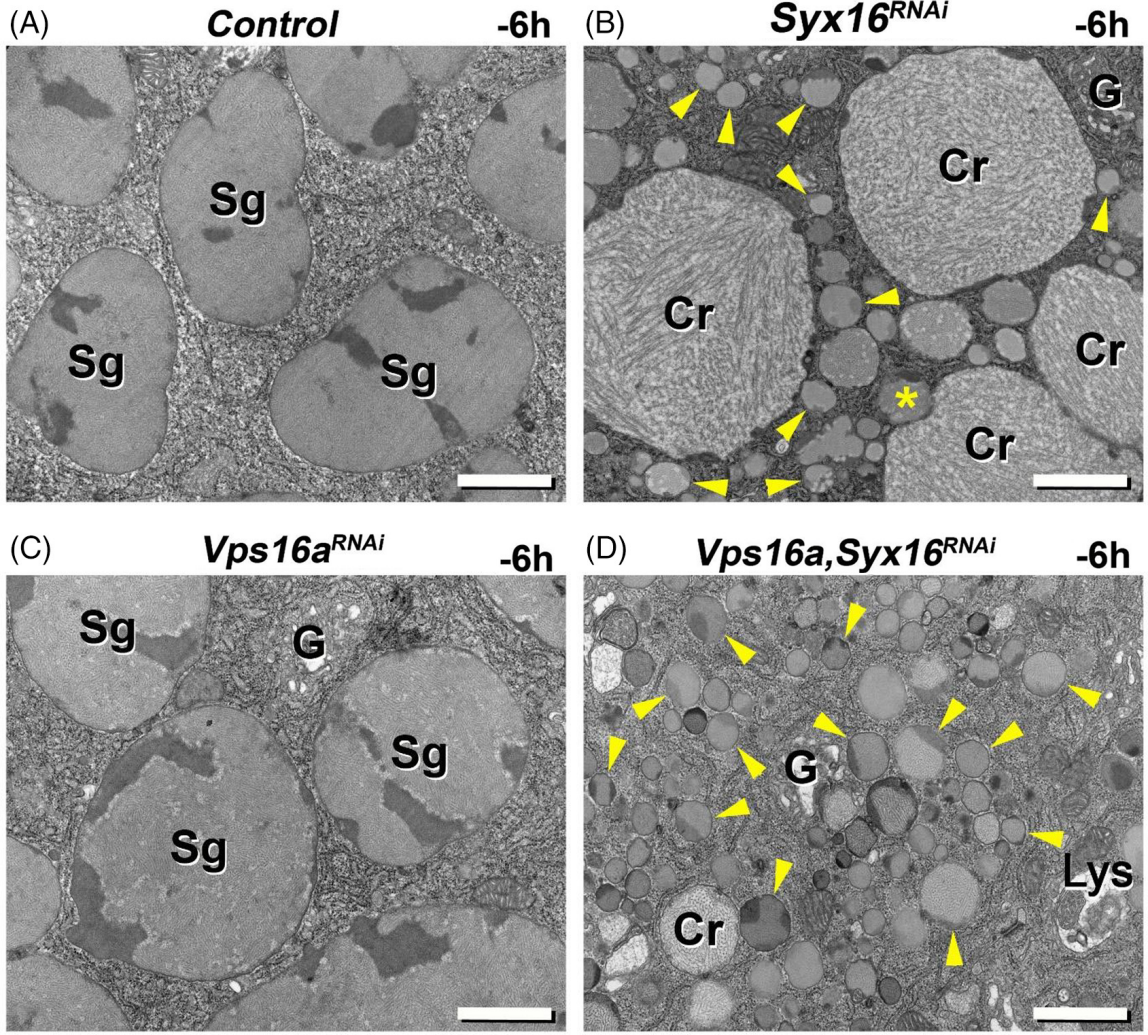
Ultrastructure of salivary gland cells from control (A), *Syx16* RNAi (B), *Vps16a* RNAi (C) and *Vps16a*, *Syx16* double RNAi (D) silenced wandering larvae. (A) Salivary gland cells from control animals contain large glue granules with normal appearance. (B) Cytoplasm of *Syx16* RNAi cells is filled with intact but very small, immature glue vesicles (yellow arrowheads) and with large crinosomes containing fibrous, electron‐lucent material. Yellow asterisk indicates fusion of immature glue granule with a crinosome. (C) In contrast, large glue granules with normal morphology are also detected in the *Vps16a* RNAi cells. (D) Simultaneous loss of function of *Vps16a* and *Syx16* results in the almost exclusive presence of immature, small glue vesicles (yellow arrowheads): there are only a few, abnormally small‐sized crinosome‐like structures with electron‐lucent fibrous content. Abbreviations: Cr–Crinosome, G–Golgi apparatus, Sg–Secretory granule, Lys – Lysosome. Bars: 1 μm (A–D)

Degradation of excess secretory granules in *Drosophila* late larval salivary gland is developmentally programmed and occurs at white prepupal stage. We were curious how the developmental program‐dependent and ‐independent glue granule degradation mechanisms interact with each other in these cells. As described above, control salivary gland cells of wandering larvae (−6 h RPF) were found to be filled with normal‐sized, intact (both Glue‐GFP and Glue‐DsRed‐positive) glue granules (Figures 1C and 5A). Moreover, these granules showed normal ultrastructural morphology and intact appearance (Figures 4A and 6A). Later, in prepupal stage (0 h RPF), these salivary gland cells contained mostly Glue‐DsRed‐positive and Glue‐GFP‐negative, thus acidic, structures, supposedly crinosomes (Figures 1D and 5B). These results suggest that the majority of the remaining glue granules was degraded via developmentally induced crinophagy.

As it was earlier mentioned, deficiency in retrograde traffic from endosomes to the TGN leads to strong accumulation and subsequent intensive early acidification and degradation of small immature secretory granules at the wandering stage of development (Figure 2B–F). As presented above, loss of function of the Qa SNARE, Syx16 causes appearance of small immature and intact glue granules, together with large, acidic crinosome‐like structures in the silenced cells (Figures 2B and 5C). The ultrastructural analysis also certified the presence of small intact glue granules and large crinosomes with loose and electron‐lucent content in the Syx16 RNAi‐treated cells (Figures 4B and 6B). Interestingly, later at the prepupal developmental stage, silencing of this gene leads to the disappearance of almost all small and intact glue granules from the cytoplasm. This observation indicates the increasing activity of secretory granule‐lysosome fusion in time, and strong cooperation of the developmental program‐dependent and ‐independent crinophagy (Figure 5D).

Absence of Vps16a, one of the subunits of the HOPS tethering complex (also required for crinophagy) caused normal glue granule size and light microscopic morphology at ‐6 h RPF stage (Figure 5E). Ultrastructural morphology of these granules was also normal, similarly to the control (Figure 6C). Compared to the control and Syx16 RNAi‐treated samples, in cells of Vps16a RNAi‐expressing prepupal animals strong glue granule acidification and degradation defects were observed (Figure 5F), as earlier mentioned.^12^

Interestingly, when Vps16a and Syx16 were silenced simultaneously, cells isolated from both the wandering and prepupal stages contained mainly intact, small glue granules and only a few but abnormally tiny acidic crinosomes (Figures 5G, H and 6D). It is important to note that, depletion of Vps16a may accumulate autophagosomes in the cytoplasm,^56^ even so the immature small glue granules never occur inside of autophagosomes in Vps16a‐Syx16 double RNAi salivary gland cells.

Moreover, we silenced simultaneously Atg1 (the key member of the initiation complex of autophagosome formation^43^) and Syx16, which showed appearance of small immature and intact glue granules, and large, acidic crinosome‐like structures in the silenced cells (Figure S2B), similar to the Syx16 RNAi (Figures 2B and 5C), compared to the control (Figures 1C, 2A, 5A, S1A and S2A) from the wandering stage of development. This observation further confirms our model, according to which during the endosome‐to‐TGN deficiency, the immature, small glue granules are degraded via direct fusion with lysosomes (crinophagy), instead of incorporation into forming autophagosomes during the macroautophagic pathway.

Taken together, these phenomena further support our model which suggests that when the endosome‐to‐TGN retrograde trafficking pathway is damaged in the *Drosophila* salivary gland cells, immature glue granules are degraded in a cooperative way: firstly, premature crinophagy then by the normal, developmentally programmed crinophagy process.

### 2.6 Endosome‐to‐TGN retrograde transport deficiency causes strong secretory defect in salivary gland from the late larval period of *Drosophila*

**FIGURE 7:**
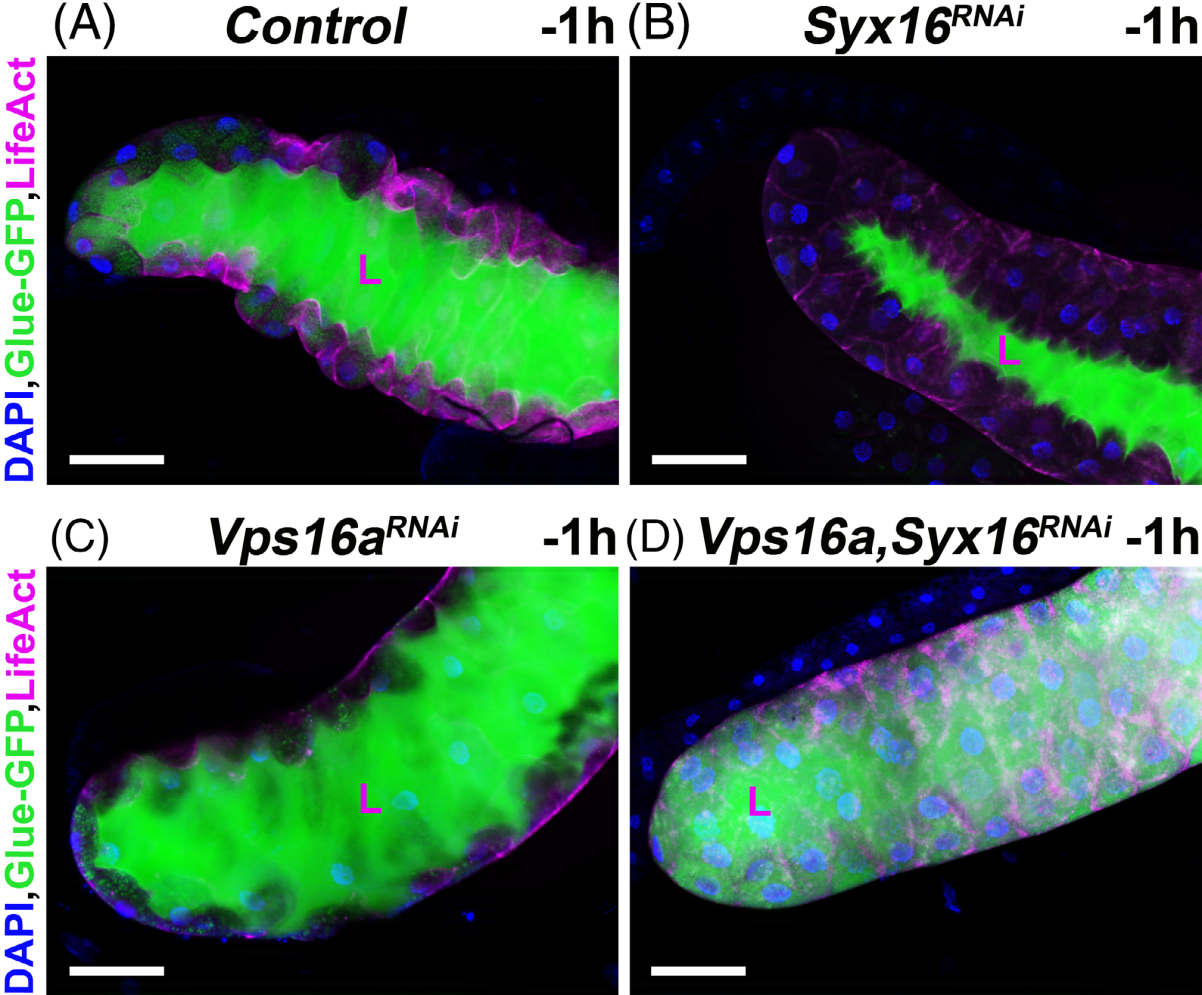
Secretory defect in the endosome‐to‐TGN retrograde transport‐deficient salivary gland cells in *Drosophila*. After the burst of glue secretion (~ −1 h RPF), the lumen of the salivary gland from control (A) and *Vps16a* RNAi (C) larva is filled with Glue‐GFP, and, in parallel, the cells become flattened. Compared to control and *Vps16a* RNAi silencing of *Syx16* (B) result in a strong secretory defect. The lumen of salivary glands from treated animals contains much less Glue‐GFP and the shape of the gland cells remains the original (as before secretion). Interestingly, silencing of *Vps16a* and *Syx16* (D) together the lumen of these salivary glands also shows much less Glue‐GFP, and the shape of the gland cells also remains normal. Furthermore, the cells still contain very small, intact (Glue‐GFP‐positive) immature glue granules. The shapes of the cells are revealed by the actin cytoskeleton marker LifeAct‐Ruby. L–lumen, scale bars: 100 μm.

The merocrine secretion requires normal endocytic activity and endosome‐to‐TGN retrograde transport mechanisms. Based on earlier results, we hypothesized that, the disruption of the endosome‐to‐TGN retrograde transport process might cause secretory defect in the salivary gland of *Drosophila*. To examine glue secretion in this tissue, we used our Glue‐GFP fusion construct described above.^10^ Moreover, we also followed cell shape changes during merocrine secretion by the transgenic actin cytoskeleton marker LifeAct‐Ruby.^35^ In control animals, lumen of the salivary gland was completely filled with Glue‐GFP, and shape of the LifeAct‐Ruby expressing cells was found to be flat indicating a successful secretory process at the late larval‐prepupal transition developmental stage (−1 h RPF) (Figure 7A). Compared to the control sample at the same developmental stage, absence of the endosome‐to‐TGN retrograde transport component Syx16 showed much less glue protein in the lumen, and the shape of cells remained unchanged (Figure 7B). Importantly, silencing of Vps16a involved in crinophagy did not show any effects in the secretory process of glue (Figure 7C). However, when Vps16a and Syx16 were silenced simultaneously, the lumen of the salivary glands contained much less glue protein, and the shape of these cells remained unchanged compared with control and Vps16a RNAi‐treated samples at the late larva to prepupa transition developmental stage (−1 h RPF). Moreover, these cells were filled with very small and intact Glue‐GFP positive immature glue granules, which indicates the failure of crinophagic degradation of these granules (Figure 7D).

Taken together, these results strongly support the role of the endosome‐to‐TGN retrograde transport mechanism in normal secretory process in the larval salivary gland cells of *Drosophila*.

### 2.7 Developmental program‐independent glue granule degradation is launched early in Vps53‐silenced salivary gland cells of *Drosophila*

**FIGURE 8:**
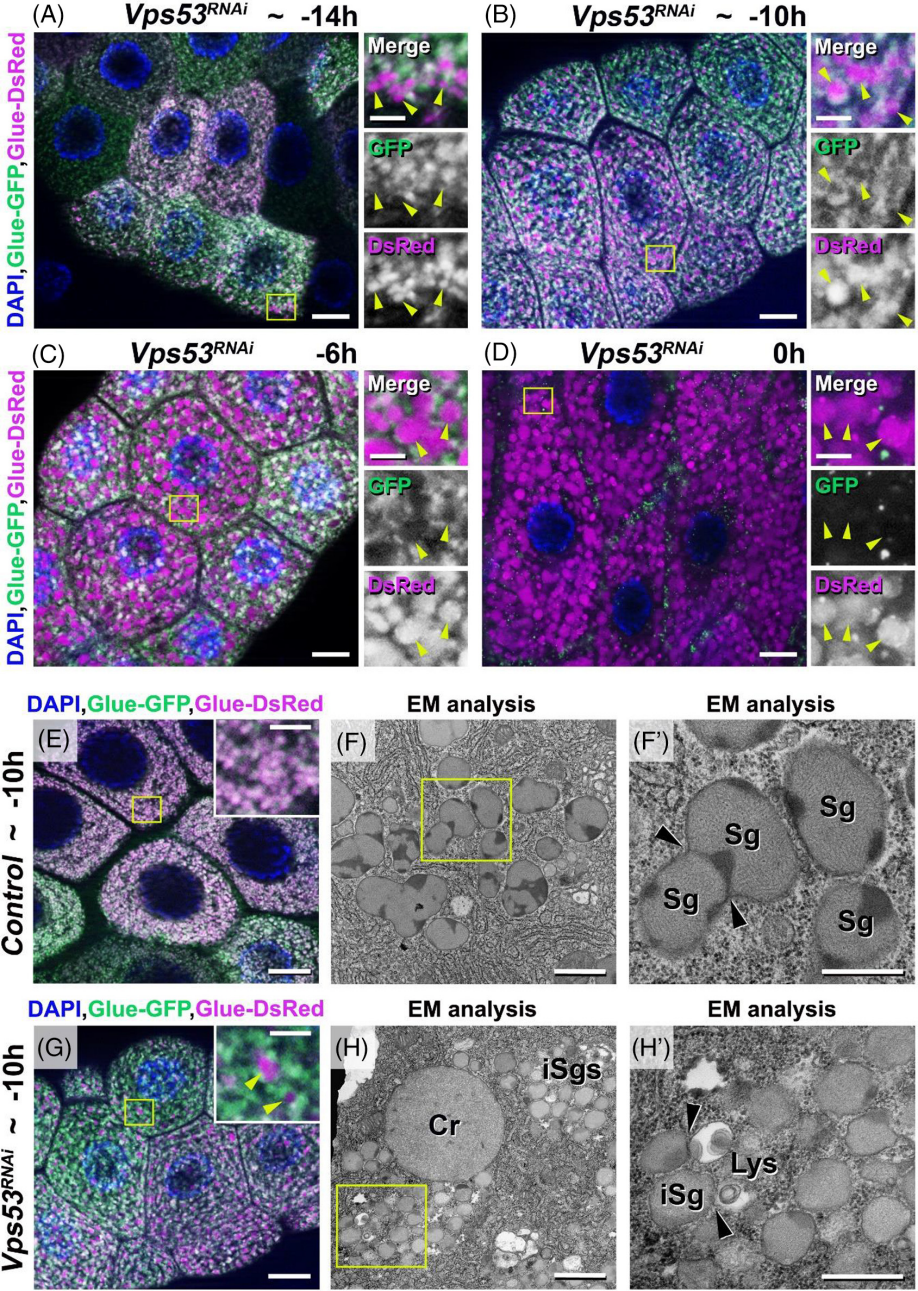
In the absence of *Vps53*, secretory granule degradation is launched early, independently from the normal developmental program. (A–D) From birth to death of glue granules in *Vps53*‐silenced salivary glands of animals co‐expressing Glue‐GFP and Glue‐DsRed (GlueFlux) reporters. (A) Early L3 stage (~ −14 h RPF) salivary gland cells contain very small (0.1–0.5 μm), mostly intact (GFP and DsRed‐positive) pre‐secretory vesicles (primordia) and larger (1.5–2.5 μm), acidic structures. (B) Mid‐L3 stage (~ −10 h RPF) salivary gland cells still include very small intact pre‐glue granules and even larger (2.5–4 μm), only DsRed‐positive crinosome‐like structures. (C) Wandering L3 stage (−6 h RPF) salivary gland cells are filled with huge (6–8 μm), acidic, DsRed‐positive granules, and they still contain a few very small (0.1–0.5 μm), intact pre‐glue granules positive for both Glue‐GFP and Glue‐DsRed. (D) In the salivary gland cells of white prepupal (0 h RPF) animals, there are even more large DsRed‐positive structures. The very small, intact glue granules (positive for both GFP and DsRed) are rarely seen. The boxed regions in panels A‐D are shown enlarged on the right side of each panel, with green and magenta channels of merged images separately, as indicated. Yellow arrowheads indicate only DsRed‐positive crinosome‐like structures. (E‐H') Comparison of fluorescent and ultrastructural data from middle L3 developmental stage (~ −10 h RPF) control and *Vps53*‐silenced animals. (E) Control salivary gland cells are filled with 0.5–1 μm, intact (Glue‐GFP‐ and Glue‐DsRed‐positive), clustering, immature glue granules. (F) Ultrastructure of the aggregated small, immature glue granules show normal morphology and size in the control sample. (F′) The boxed region of panel F is shown enlarged in F′, where the black arrowheads indicate the homotypic fusion of immature glue granules. (G) Compared to control, *Vps53*‐silenced gland cells contain much less intact pre glue granules but some larger acidic (only Glue‐DsRed‐positive) structures are also present. (H) Ultrastructural analysis of *Vps53*‐silenced cells shows aggregated, small glue granule primordia and some crinosomes. (H′) The boxed region of panel H is shown enlarged, where black arrowheads point to the heterotypic fusion of pre‐glue granules with lysosome‐like structures. The boxed regions in panels, E and G are shown enlarged on the right side of each panel. Yellow arrowheads indicate only DsRed‐positive crinosome‐like structures in panel G. Abbreviations: Cr–Crinosome, iSg–immature Secretory granule or primordium, Lys–Lysosome, Sg–Secretory granule. Bars: 20 μm (A–D, E and G), 5 μm (A–D, E and G insets), 1 μm (F and H), 0.5 μm (F′ and H′)

To decide whether early crinophagy of the small immature glue granules in case of retrograde transport deficiency is caused by the secretory defect described above or other phenomenon, we have chosen Vps53, as a representative component of the retrograde transport mechanism and examined the Vps53 RNAi phenotype with the “GlueFlux” system in several developmental stages (Figure 8A–D). At the early L3 stage (~ −14 h RPF) control, non‐silenced salivary gland cells contained very small glue granule primordia (Figure 1A) while silencing of Vps53 resulted in appearance of not only intact (Glue‐GFP and Glue‐DsRed‐positive) granule primordia, but also larger, acidic (positive only for Glue‐DsRed) crinosome‐like structures (Figure 8A). Moreover, these crinosomes became even larger and more numerous in course of development (Figure 8B–D) compared to the control (Figure 1B–D). Importantly, while the size of these intact granule primordia was not changed, their number decreased when the Vps53 RNAi‐treated animal reached the prepupa stage (0 h RPF). This phenomenon suggests that crinophagy in Vps53 silenced cells is induced at a very early developmental stage, independently form the developmental program and the secretory defect.

Next, we examined the ultrastructure of salivary gland cells at the middle L3 developmental stage (~ −10 h RPF) in control and Vps53‐silenced tissues and compared them with our previous observations using the “GlueFlux” system. As presented above, in normal cells, there were a lot of aggregated immature secretory vesicles (0.5–1 μm), which were positive for both Glue‐GFP and Glue‐DsRed (Figures 1B and 8E). These glue granules showed normal ultrastructure and were similar to mature granules present in a later wandering stage, except for their size (Figure 8F). However, at this stage of development, aggregated immature glue granules were detected to undergo homotypic fusion, just creating the final‐size, mature secretory granules (Figure 8F'). Compared to the control, Vps53 deficiency caused the accumulation of glue granule primordia (0.1–0.5 μm), which were mainly intact (Glue‐GFP and Glue‐DsRed‐positive). In addition, acidic (only Glue‐DsRed‐positive) crinosome‐like structures, normally not present at the middle developmental stage also appeared (Figure 8B, G). Ultrastructure of these Vps53 RNAi treated cells revealed aggregated, small granule primordia and large crinosome‐like structures (Figure 8H). In contrast to control, these aggregated glue granule primordia were not seen to fuse with each other, although they underwent heterotypic fusion with lysosome‐like structures, thus forming crinosomes (Figure 8H').

These observations point that disruption of the endosome‐to‐TGN retrograde transport causes early defects in glue granule homotypic fusion, and these accumulated granule primordia fuse preferably with lysosomes. This process takes place even during the initial steps of glue granule formation, independently from and preceding the developmental program and manifestation of the secretory defect.

### 2.8 Localization of the endosome‐to‐TGN retrograde transport component Synaptobrevin (Syb) in the salivary gland cells at different developmental stages

Many of the molecular components which coordinate glue granule homotypic fusion during granule maturation, such as SNAREs, tether factors and small GTPases are still unknown.

**FIGURE 9:**
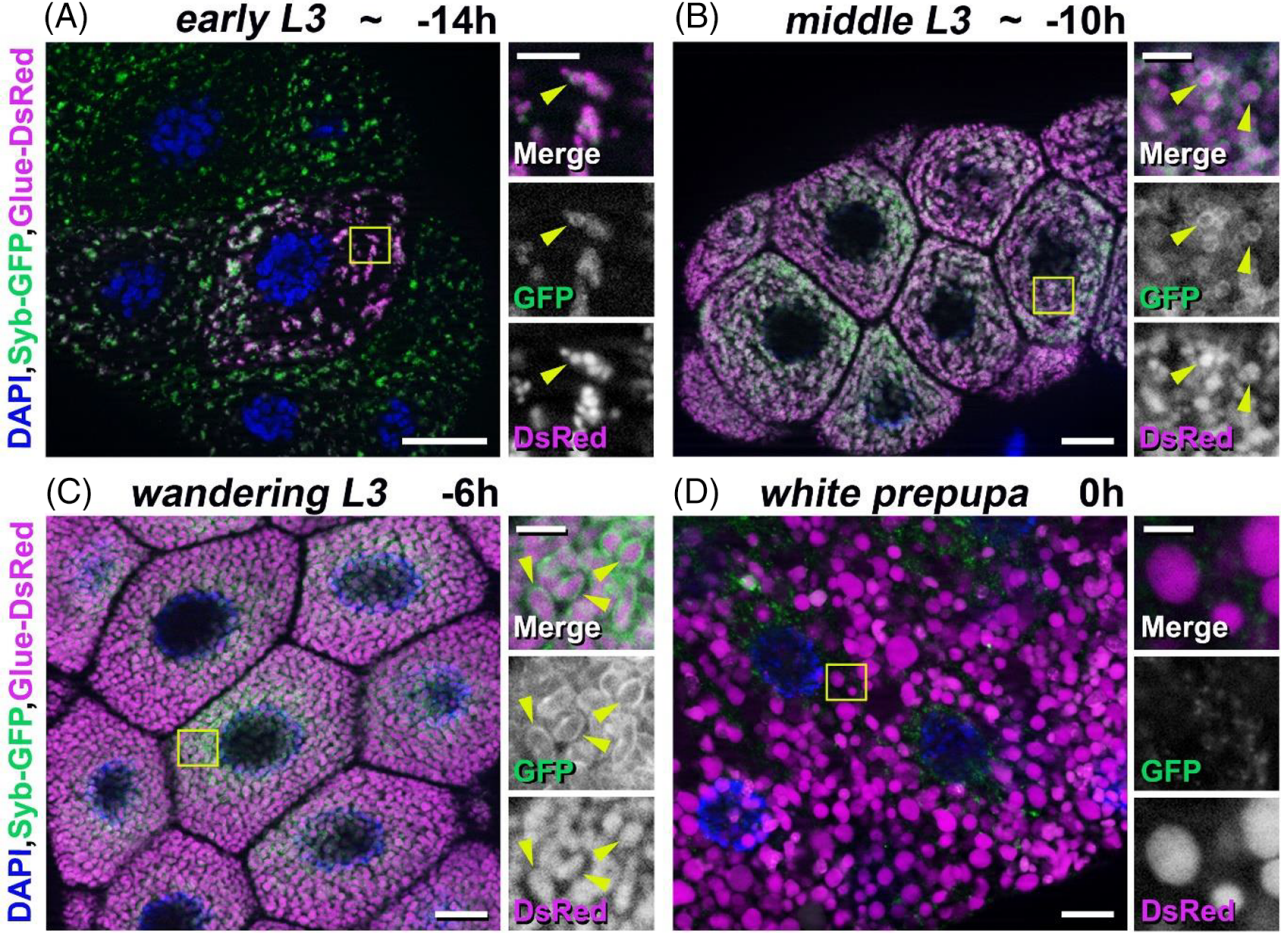
Time‐course of Synaptobrevin (*Syb*) localization during four developmental stages. (A) During initiation of glue granule formation (early L3 developmental stage, ~ −14 h RPF), the GFP‐tagged Synaptobrevin (*Syb*) is already localized to the membrane of forming glue granule primordia (yellow arrowheads). (B–C) At later stages (~ −10 h and ‐6 h RPF), these granules are still positive for Syb‐GFP. (D) During secretory granule‐lysosome fusion, Syb‐GFP is already absent from the membrane of degrading glue granules. Yellow arrowheads indicate the localization of Syb‐GFP in the membrane of granule primordia (A), immature (B) and mature (C) glue granules in the right insets. The boxed regions in panels A‐D are shown enlarged on the right side of each panel. Green and magenta channels of merged images are also shown separately as indicated. Bars: 20 μm (A–D), 5 μm (A–D right insets)

Based on earlier data and our own results, we hypothesized that the R‐SNARE protein, Syb may be part of the SNARE complex coordinating granule‐to‐granule fusion in *Drosophila* larval salivary gland cells at the early (~ −14 h RPF) and middle (~ −10 h RPF) developmental stages. Therefore, we examined the localization of a GFP tagged Syb in the gland cells. As the development of glue granules started at the early stage, some cells contained small glue granule primordia with membrane already positive for Syb‐GFP (Figure 9A). In contrast, salivary gland cells of middle stage (~ −10 h) contained larger, immature and aggregated glue granules and Syb‐GFP also formed small rings around the Glue‐DsRed‐positive granules (Figure 9B). Later at the wandering stage of development, salivary gland cells were filled with large, mature glue granules which were ready for exocytosis. Importantly, the membrane of these granules was still positive for Syb‐GFP, indicating an essential role for Syb in merocrine secretion (Figure 9C). Interestingly, after the burst of secretion and then crinophagy in prepupal stage, when nothing but degrading glue granules are present in salivary gland cells, Syb‐GFP was not present in the membrane of these structures (Figure 9D).

These results indicates that, Syb might be a part of the membrane fusion apparat, which coordinates granule‐to‐granule fusion in *Drosophila* larval salivary gland cells during the development.

## 3 DISCUSSION

Formation and maturation of secretory granules containing mucopolysaccharide‐type glue proteins in the larval salivary gland cells of fruit fly are developmentally programmed. They require several steps of vesicular trafficking pathways, especially the formation and homotypic fusion of glue granule primordia, thus creating large mature secretory granules ready for exocytosis.^11^ Our observations indicate that in larval salivary gland cells neither primordial and immature glue granules nor the matured, large secretory granules are acidic (Figure 1A–C), in contrast with samples from prepupal animals (Figure 1D). Importantly, using the “GlueFlux” system, we have shown earlier that at this developmental stage, the acidic (only DsRed‐positive) glue granules are degradative structures resulted from direct fusion with late endosomes or lysosomes (via crinophagy).^12^ Taken together, glue‐containing secretory granule primordia start to form already at the early L3 stage of development and get larger via homotypic fusion, thereby creating larger immature secretory granules. Then, the aggregation and further homotypic fusion of these immature granules end in full‐sized mature glue granules already suitable for secretion. Glue granules avoiding exocytosis eventually turn acidic and degrade via crinophagy.

Earlier studies identified several factors essential for formation and homotypic fusion of these granules, such as clathrin and its adaptor AP‐1 complex, the AP‐1 recruiting small GTPase, Arl1 and the lipid kinase, PI4KIIα.^11^, ^24^, ^33^ To extend our knowledge concerning their function in gland cells, we examined the possible role of these factors in glue granule maturation and early degradation with our “GlueFlux” system. We found that loss of function of Arl1, AP‐1γ, clathrin heavy chain (Chc) and PI4KIIα equally causes insufficient glue granule maturation, intensive early acidification and degradation of glue in *Drosophila* salivary gland cells preceding pupariation, at the wandering larval stage of development (Figure S1A–E).

Increased Ca^2+^ concentration is necessary not only for regulation of exocytosis, but also important in clathrin‐mediated endocytosis (CME). Ca^2+^ also has a function in the reformation of synaptic vesicles in *Drosophila* larval neuromuscular junctions. The *Drosophila* flower (Fwe) transmembrane protein, in a multimer form, localizes to the surface of synaptic vesicles, and can create a channel to regulate Ca^2+^ influx.^57^, ^58^ Based on the similar function of secretory granules and synaptic vesicles, we silenced the Fwe gene in the larval salivary gland of *Drosophila*, and we found that the loss of function of Fwe also caused defects in glue granule maturation, intensive early acidification and degradation of immature glue granules (Figure S1F). These observations may be in connection with the possible role of Fwe in the endocytotic and glue granule homotypic fusion processes required for secretory granule maturation.^25^, ^57^

SNARE proteins are also necessary for secretory granule maturation and homotypic fusion. Based on localization data in *Drosophila* larval salivary gland cells role of the Qbc‐type SNARE, Snap24 (fruit fly orthologue of the mammalian Snap23 and Snap25), as a putative mediator of granule‐to‐granule fusion was described.^15^ We screened all of the known *Drosophila* SNAREs using the “GlueFlux” system, and in contrast with these observations, our results indicate that silencing of Snap24 in these cells has no effect on glue granule maturation and homotypic fusion and does not cause early crinophagic degradation of these granules (Table S1). Furthermore, it was shown that Tlg1 (yeast orthologue of the Qc‐type SNARE, Syx6) in strong cooperation with the Qa‐type Tlg2 (Syx16 orthologue in yeast) has a function in TGN‐vesicle homotypic fusion in *Saccharomyces cerevisiae*.^59^ Syx6 is found to be necessary for immature secretory granule homotypic fusion in PC12/PC2 cell cultures as well, and it is also involved in endocytic and secretory pathways in pancreatic β‐cell cultures.^60^, ^61^ Moreover, Vti1a, the other TGN‐related Qb‐type SNARE also has a function in secretory granule biogenesis in adrenal chromaffin cells.^62^ Even so, our results in larval salivary gland cells of *Drosophila* show that, in contrast to Syx16, loss of function of Syx6 and Vti1a has no effect on glue granule homotypic fusion and maturation and does not cause early acidification and degradation of these granules (Table S1). A possible explanation is that in this species there are other Qb, Qc, or Qbc‐type SNAREs with supplementary function in secretory granule maturation and granule‐to‐granule fusion processes.

On the other hand, via membrane recycling, early endosomal contribution and retrograde transport from the early endosomal compartment to the TGN are also required in secretory granule homotypic fusion and maturation. For example, SNAREs for granule‐to‐granule fusion and lipids from the early endosomal compartment to the TGN were found to be needed to maintain glue granule maturation.^11^, ^25^, ^33^ Importantly, Ma et al. earlier identified several early endosome‐derived and retrograde transport‐connected factors, such as the above‐mentioned Syx16, members of the GARP tethering complex and the TGN‐related small GTPase, Rab6 which are all involved in glue granule maturation process.^25^ Moreover, several studies clearly show that absence of the endosome‐to‐TGN retrograde trafficking strongly perturbs the secretory process and directs proteins and lipids for lysosomal degradation.^27^, ^37^, ^38^, ^39^, ^63^ Notably, these factors are important research objects in this present study as well and were proven to also have a function in avoiding immature secretory granules from early acidification and crinophagic degradation.

In this study, we showed that loss of function of Syx16, Syb, Rab6, or the key members of the GARP tethering complex (Vps53 and Vps54) caused not only secretory granule formation and maturation failure, but also led to premature crinophagic degradation of the immature forms of glue granules (Figures 2A–F, 3A–F and 4A–F). Interestingly, defects of the retrograde trafficking pathway resulted in strong accumulation of large, acidic and prematurely formed glue‐containing crinosomes in the cytoplasm of the RNAi‐treated gland cells. Based on our earlier published work about the molecular mechanisms of developmentally programmed crinophagy,^12^ we concluded that these structures that can be detected as Glue‐DsRed‐positive (acidic) granules in fluorescent micrographs (Figure 2B–F) are presumably crinosomes. It is feasible that these structures were formed via crinophagic degradation of immature granules, firstly, because some immature glue granule membranes were found to be decorated with GFP‐tagged lysosomal membrane protein (GFP‐Lamp1) hallmark of crinophagy (Figure 3B–F). As a mentioned earlier, the large crinosome‐like structures were not positive for GFP‐Lamp1, because in this construct, the GFP part faced to the luminal side and was rapidly quenched in acidic lysosomes and crinosomes.^12^, ^55^ Moreover, crinosomes get larger because of increased crinophagic activity and/or homotypic fusion of these crinosomes.

Secondly, ultrastructure of these large acidic compartments (Figure 4B–F) is similar to that of the crinosomes described earlier.^12^ Thirdly, when we simultaneously silenced the TGN‐related retrograde transport factor, Syx16 and the nonspecific HOPS tethering complex component, Vps16a (previously shown to be required for crinophagy^12^), these large and acidic structures were not further observed, only immature but non‐acidic small glue granules were detected in the salivary gland cells of both wandering and prepupal developmental stages (Figures 5G, H and 6D). Importantly, Syx16 and Vps16a are not required for microautophagy, but in addition of crinophagy, Vps16a is necessary for autophagosome‐lysosome fusion too.^56^, ^64^ During ultrastructural analysis of the salivary gland cells containing Vps16a‐Syx16 double RNAi, we experienced that, these cells never included immature and small glue granules inside of their autophagosomes. Moreover, when we silenced simultaneously Atg1 (which is required for autophagosome formation) and Syx16, salivary gland cells still contained immature and intact small glue granules together with large acidic crinosome like structures (Figure S2 A and B), similar to Syx16 RNAi. Consequently, macro‐ and microautophagy do not contribute to the initial step of the formation of large and acidic crinosomes in salivary gland cells in which the endosome‐to‐TGN retrograde trafficking pathway is absent. Moreover, we have earlier identified that, macroautophagy is not necessary for the developmentally programmed glue granule‐lysosome fusion in the late larval salivary gland cells of *Drosophila*.^12^ Finally, ultrastructural analysis of the Vps53‐silenced salivary gland cells in the middle stage of development clearly revealed immature glue granule‐to‐lysosome fusions (Figure 8H, H') instead of granule‐to‐granule fusion processes. These findings strongly indicate that retrograde recycling from endosomes and immature glue granules to the TGN are required for secretory granule maturation and homotypic fusion as well to avoid early aging and degradation of immature glue granules. The possible reason is that retrograde recycling of membrane proteins and lipids—required for granule‐to‐granule fusion—among early endosomal, immature secretory granule as well as TGN compartments are necessary for maintaining this maturation process in *Drosophila* as well.^25^, ^33^, ^65^ Because complete maturation can inhibit early crinophagic degradation of the immature forms of glue granules, it is possible that crinophagy acts as a secretory granule size and/or quality control mechanism which, when needed, is activated at very early stages of development and removes abnormal glue granules from the cytoplasm preventing their exocytosis independently of the normal developmental program (Figure 8A, B, G, H and H').

Interestingly, loss of function of Rab6 causes defects not only in the retrograde transport, but also perturbs the insulin receptor recycling mechanisms.^66^ Based on our ultrastructural results, latter situation may contribute to the activation of macroautophagy which can account for the high amount of autophagic structures in the Rab6‐RNAi salivary gland cells (Figure 4D).

After the burst of glue secretion, the lumen of the *Drosophila* salivary gland is fully filled with glue proteins. In parallel, shape of the gland cells changes drastically they get flattened which is an important step of the normal glue expulsion and required for filling the lumen with a large amount of glue.^67^ Interestingly, earlier studies showed that deficiency of the endosome‐to‐TGN retrograde transport caused strong defects in secretory mechanisms as well.^37^, ^38^, ^39^ We also found that, failure of the endosome‐to‐TGN retrograde trafficking led to defects of glue secretion in the late larval salivary glands, moreover, shape of the gland cells remained unchanged (Figure 7B, D). It is important to note that failure of merocrine secretion often causes intensive secretory granule degradation which is accomplished mainly by crinophagy.^8^, ^48^ Importantly, we also checked the effects of Vps16a RNAi and Vps16a;Syx16 double RNAi in the secretory process. Vps16a is required for crinophagy, but not for secretion of glue (Figure 7C). However, silencing of Vps16a and Syx16 together causes strong secretory defect, indeed the immature glue granules have not been removed by crinophagy from the cells (Figure 7D). Furthermore, degradation of immature granules occurs already at early and middle developmental stages in the Vps53 RNAi cells (Figure 8A, B, G, H and H') indicating that this early induced immature granule‐to‐lysosome fusion is not connected to secretory failing, because glue is not yet secreted at the early developmental stages in *Drosophila* salivary glands.^10^ Therefore, failure of glue secretion in the endosome‐to‐TGN retrograde transport‐deficient salivary gland cells may presumably be on account of a secretory granule maturation defect and early activated crinophagy. Furthermore, this secretory defect during the late larval period in Syx16‐silenced cells also indicates that the intact, immature secretory granules and prematurely formed crinosomes (present in high levels in the retrograde transport‐deficient salivary gland cells), may not prefer to fuse with the plasma membrane, in contrast to the large and intact glue granules in control cells.

Finally, we examined the localization of the R‐type SNARE, Synaptobrevin (Syb) which have several functions in the cell. Syb is required for merocrine secretion and one of its mammalian orthologues Vamp4 is necessary also for recycling and intracellular sorting of synaptic receptors.^54^, ^68^ Moreover, the other Syb orthologues Vamp1 and Vamp2 are present on the surface of Glut4‐containing vesicles.^69^ Our results show that the GFP‐tagged Syb is already localized to the surface of glue granule primordia and immature glue granules at very early in the L3 developmental stages (~ −14 and −10 h RPF) which indicates a possible role of this SNARE protein in the homotypic fusion of glue‐containing vesicles (Figure 9A, B). The role of Syb in glue exocytosis is proved by the localization of Syb‐GFP on the membrane of mature, large glue granules at the wandering L3 stage (−6 h RPF); however, this protein is missing from the majority of the crinosomal membranes mainly formed after the burst of secretory process at the prepupal stage of development (Figure 9C, D). Importantly, the loss of function of Syb causes strong accumulation of immature glue granules in the cytoplasm, and a subsequent early crinophagic degradation of these structures (Figures 2C, 3C and 4C). These results suggest that Syb is not only necessary for exocytosis and the endosome‐to‐TGN retrograde trafficking, but also may have a role in the homotypic fusion of the immature glue granules during the maturation process. Moreover, crinosomes, which are usually not secreted by exocytosis, were found to be not positive for Syb‐GFP further supports the role of this protein in the merocrine secretion of glue in the larval salivary gland of *Drosophila*. In summary, Syb might be the part of the SNARE complex which not only mediates glue granule exocytosis, but also might be required for endosome‐to‐TGN retrograde trafficking and granule‐to‐granule fusion in the salivary gland cells of *Drosophila* during the development.

Importantly, the wild type form of Rab6 was also identified on the membrane of secretory granules in exocrine pancreas, atrial myocytes and larval salivary gland cells in *Drosophila* which suggest similar function in these tissues.^70^, ^71^, ^72^ This phenomenon suggests that Rab6 may be a part of the membrane fusion apparat, which directly regulates granule‐to‐granule fusion in salivary gland cells of *Drosophila*.

**FIGURE 10:**
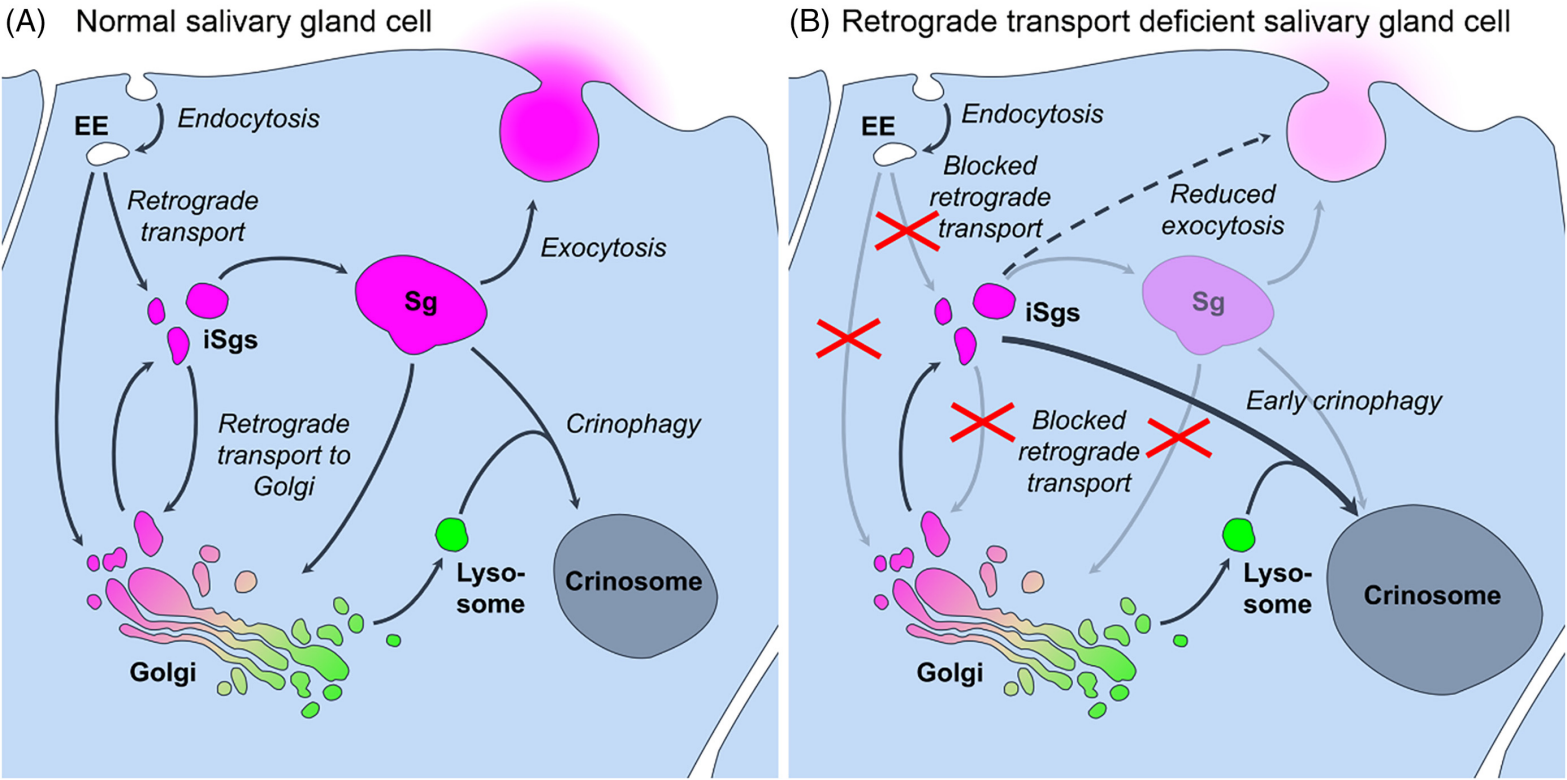
Model of the possible connections among glue granule maturation, secretion and crinophagy in normal and retrograde transport‐deficient salivary gland cells of *Drosophila*. (A) In normal salivary gland cells, retrograde transport from endosome‐to‐TGN and from secretory granule‐to‐TGN contribute to secretory granule growth and maturation, prerequisite for their exocytosis or developmentally programmed crinophagy. (B) Blocked retrograde transport mechanisms cause a failure in secretory granule maturation and result in cytoplasmic aggregation, reduced exocytosis and premature crinophagic degradation of immature secretory granules independently from the developmental program. Abbreviations: EE–Early endosome, iSg–immature Secretory granule, Sg–Secretory granule.

Taken together, in the absence of retrograde transport mechanisms, glue granules cannot reach their normal size because of the failure of recycling of membrane proteins and lipids necessary for glue granule homotypic fusion. Therefore, the accumulating abnormally small glue vesicles become degraded by crinophagy activated before normal time (Figure 10). This presently described, developmental program‐independently, prematurely activated crinophagy may add to the knowledge of the molecular mechanisms by which secretory granules are targeted for crinophagic degradation instead of exocytosis. Our work hopefully contributes to a better understanding of the connections among merocrine secretion and secretory granule homotypic fusion, maturation and degradation and paves the way for further investigation of these links in animal models and humans.

## 4 MATERIALS AND METHODS

### 4.1 Fly stocks and work

The following fly stocks were obtained from the Bloomington *Drosophila* Stock Center: *Sgs3* (*Glue*)*‐GFP*,^10^ *UAS‐LifeAct‐Ruby*,^35^ *UAS‐Syb‐GFP* and RNAi stocks generated by the Transgenic RNAi Project including *UAS‐Syx16* ^*JF01924*^, *UAS‐Rab6* ^*JF02640*^, *UAS‐Vps53* ^*HMS01712*^, *UAS‐Vps54* ^*HMS01910*^, *UAS‐AP‐1γ* ^*JF02684*^, *UAS‐Arl1* ^*JF02378*^ and *UAS‐Fwe* ^*JF02632*^. *UAS‐Syb* ^*KK113351*^ and *UAS‐Atg1* ^*GD16133*^ RNAi lines were obtained from the Vienna *Drosophila* Resource Center, while the *UAS‐Chc* ^*9012R‐1*^ *and UAS‐PI4KIIα* ^*2929R‐3*^ RNAi lines were from the National Institute of Genetics of Shigen, Japan (Nig‐Fly). Additional fly lines were provided as listed below: *UAS‐GFP‐Lamp1* and *UAS‐Vps16a* ^*RNAi*^ by H. Krämer, Center for Basic Neuroscience, UT Southwestern Medical Center^55^; *Sgs3* (*Glue*)*‐DsRed* by A. Andres, University of Nevada, Las Vegas, NV^53^; and *fkhGal4* by E. Baehrecke, University of Massachusetts Medical School, Worcester, MA.^50^ In this study for examination of crinophagy we used the earlier developed fly constructs *Glue‐GFP, Glue‐DsRed; fkhGal4* and *Glue‐DsRed, UAS‐GFP‐Lamp1; fkhGal4*.^12^

### 4.2 Fluorescent microscopy

Salivary glands were dissected from control and RNAi animals at the indicated developmental stages, fixed briefly (for 5 minutes) in 4% paraformaldehyde in PBS (pH 7.4), and covered with PBS‐Glycerin (9:1) containing DAPI. Images were taken using a Carl Zeiss AxioImager M2 epifluorescent microscope using an AxioCam MRm camera and Plan‐Apochromat 63x NA = 1.4, EC Plan‐Neofluar 40x NA = 0.75 and EC Plan‐Neofluar 10x NA = 0.3 objectives and processed in Zeiss AxioVision SE64 Rel. 4.9.1 and Adobe Photoshop CS3 Extended.

### 4.3 Transmission electron microscopy (TEM)

Dissected salivary glands were fixed in 3.2% paraformaldehyde, 0.5% glutaraldehyde, 1% sucrose, 0.028% CaCl_2_ in 0.1 M sodium cacodylate buffer and pH 7.4 for overnight at 4°C. Samples were postfixed in 0.5% osmium tetroxide for 1 h and in half‐saturated aqueous uranyl acetate for 30 min, dehydrated in a graded series of ethanol and embedded into Durcupan (Fluka) according to the manufacturer's recommendations. 70 nm sections were stained in Reynold's lead citrate and viewed in a transmission electron microscope (JEM‐1011; JEOL) equipped with a digital camera (Morada; Olympus) using iTEM software 5.1 (Olympus).

## AUTHOR CONTRIBUTIONS

Tamás Csizmadia designed the research; Tamás Csizmadia, Anna Dósa, Erika Farkas, Belián Valentin Csikos, Eszter Adél Kriska and Péter Lőw performed the experiments; Tamás Csizmadia, Gábor Juhász and Péter Lőw evaluated the data and Tamás Csizmadia and Péter Lőw wrote the paper with comments from all authors.

## CONFLICT OF INTEREST

The authors declare that there is no conflict of interest.

### PEER REVIEW

The peer review history for this article is available at https://publons.com/publon/10.1111/tra.12871.

## Supporting information


**Figure S1.** Loss of function of genes involved in secretory granule formation and maturation leads to early acidification and breakdown of immature glue granules in *Drosophila* salivary gland cells at the wandering stage of development. (A‐F) Degradation of very small glue granules in the salivary gland cells of wandering L3 (−6 h RPF) animals co‐expressing Glue‐GFP/Glue‐DsRed (GlueFlux) reporters and the RNAi‐construct of the given secretory granule maturation‐connected gene. (A) Control wandering L3 stage (−6 h RPF) larval salivary gland cells normally enclose large (3–3.5 μm), intact (GFP‐ and DsRed‐double positive) glue‐containing secretory granules. Compared to the control, salivary gland cells with the loss of function of the small GTPase, *Arl1* (B), the clathrin adaptor complex subunit *AP‐1γ* (C), the clathrin heavy chain component *Chc* (D), the lipid kinase *PI4KIIα* (E) and the Ca^2+^ channel *Fwe* (F) contain very small (0.1–1.5 μm) immature, intact secretory vesicles positive for both GFP and DsRed. Interestingly, these cells also include degradative (positive for DsRed only) large (4–8 μm) acidic glue granules (crinosomes) indicated by yellow arrowheads in the right insets. The boxed regions in panels A–F are shown enlarged on the right side of each panel. Green and magenta channels of merged images are also shown separately as indicated. Bars: 20 μm (A–F), 5 μm (A–F right insets)
**Figure S2.** Silencing of Atg1 and Syx16 genes simultaneously in salivary gland cells. (A–B) Degradation of small glue granules in the salivary gland cells of wandering L3 (−6 h RPF) animals co‐expressing Glue‐GFP/Glue‐DsRed (GlueFlux) reporters and the double RNAi‐construct of Atg1 and Syx16 genes. (A) Control wandering L3 stage (−6 h RPF) larval salivary gland cells normally contain large (3–3.5 μm), intact (GFP‐ and DsRed‐double positive) glue granules. Compared to the control, salivary gland cells with Atg1‐Syx16 double RNAi cause the accumulation of immature (1–1,5 μm), intact, glue granules and large (3–6 μm), acidic structures in these cells (indicated by yellow arrowheads in the right insets) which strongly suggests premature glue degradation at wandering (−6 h RPF) stage. Bars: 20 μm (A–B), 5 μm (A–B right insets)

Table S1.
