# Supplementary material for: Developmental program‐independent secretory granule degradation in larval salivary gland cells of Drosophila
Source: Traffic. 2022 Nov 29;23(12):568–86. doi: 10.1111/tra.12871 (PMC10099382; doi:10.1111/tra.12871)
Supplement: Supplementary file 1 — Figure S1. Loss of function of genes involved in secretory granule formation and maturation leads to early acidification and breakdown of immature glue granules in Drosophila salivary gland cells at the wandering stage of development. (A‐F) Degradation of very small glue granules in the salivary gland cells of wandering L3 (−6 h RPF) animals co‐expressing Glue‐GFP/Glue‐DsRed (GlueFlux) reporters and the RNAi‐construct of the given secretory granule maturation‐connected gene. (A) Control wandering L3 stage (−6 h RPF) larval salivary gland cells normally enclose large (3–3.5 μm), intact (GFP‐ and DsRed‐double positive) glue‐containing secretory granules. Compared to the control, salivary gland cells with the loss of function of the small GTPase, Arl1 (B), the clathrin adaptor complex subunit AP‐1γ (C), the clathrin heavy chain component Chc (D), the lipid kinase PI4KIIα (E) and the Ca2+ channel Fwe (F) contain very small (0.1–1.5 μm) immature, intact secretory vesicles positive for both GFP and DsRed. Interestingly, these cells also include degradative (positive for DsRed only) large (4–8 μm) acidic glue granules (crinosomes) indicated by yellow arrowheads in the right insets. The boxed regions in panels A–F are shown enlarged on the right side of each panel. Green and magenta channels of merged images are also shown separately as indicated. Bars: 20 μm (A–F), 5 μm (A–F right insets) Figure S2. Silencing of Atg1 and Syx16 genes simultaneously in salivary gland cells. (A–B) Degradation of small glue granules in the salivary gland cells of wandering L3 (−6 h RPF) animals co‐expressing Glue‐GFP/Glue‐DsRed (GlueFlux) reporters and the double RNAi‐construct of Atg1 and Syx16 genes. (A) Control wandering L3 stage (−6 h RPF) larval salivary gland cells normally contain large (3–3.5 μm), intact (GFP‐ and DsRed‐double positive) glue granules. Compared to the control, salivary gland cells with Atg1‐Syx16 double RNAi cause the accumulation of immature (1–1,5 μm), intact, glue gr [file TRA-23-568-s002.docx]

**Supplementary figures**


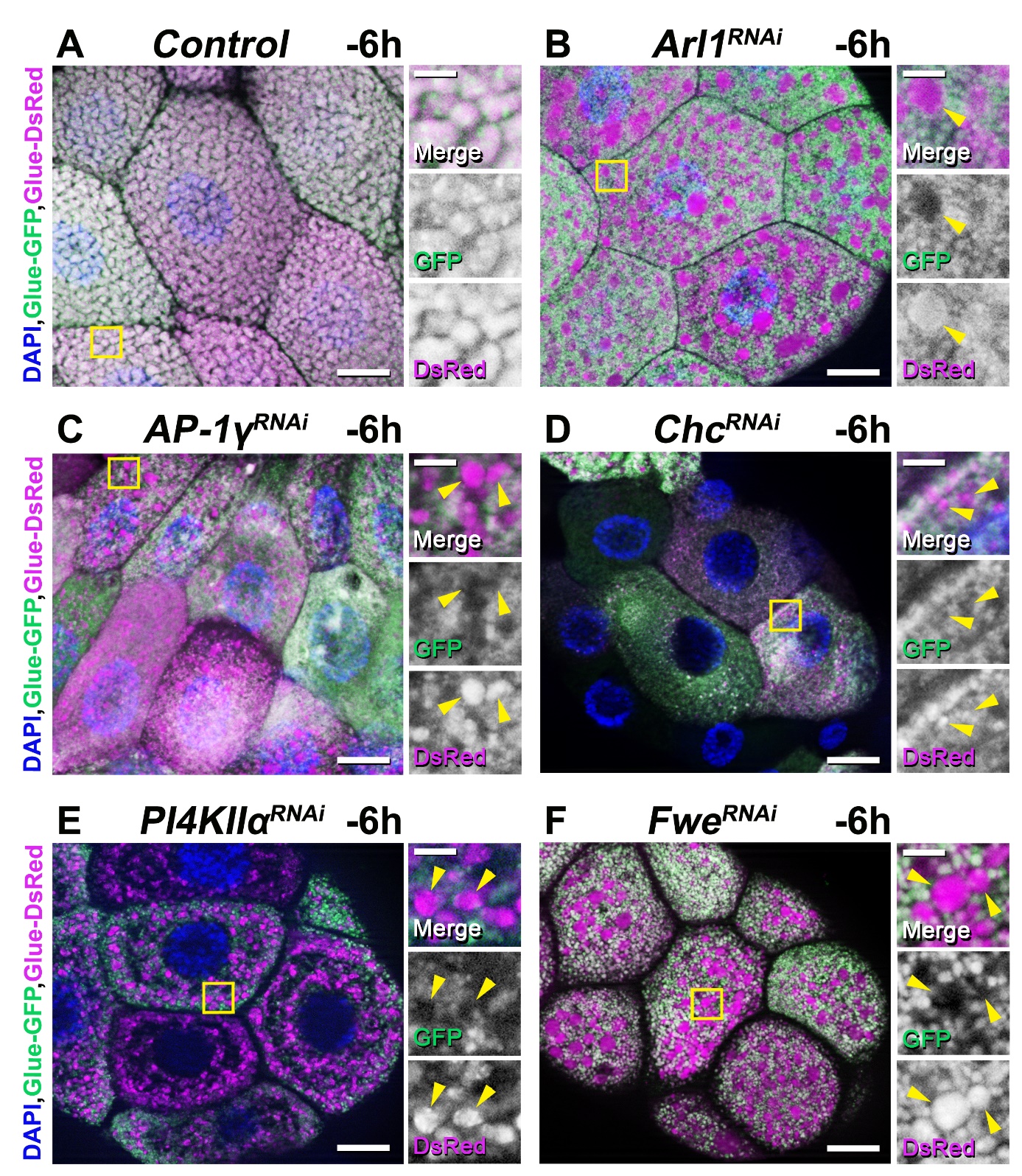


**Figure S1 Loss of function of genes involved in secretory granule formation and maturation leads to early acidification and breakdown of immature glue granules in *Drosophila* salivary gland cells at the wandering stage of development.**

(A-F) Degradation of very small glue granules in the salivary gland cells of wandering L3 (-6h RPF) animals co-expressing Glue-GFP/Glue-DsRed (GlueFlux) reporters and the RNAi-construct of the given secretory granule maturation-connected gene. (A) Control wandering L3 stage (-6h RPF) larval salivary gland cells normally enclose large (3-3.5 µm), intact (GFP- and DsRed-double positive), glue-containing secretory granules. Compared to the control, salivary gland cells with the loss of function of the small GTPase, *Arl1* (B), the clathrin adaptor complex subunit *AP-1γ* (C), the clathrin heavy chain component *Chc* (D), the lipid kinase *PI4KIIα* (E) and the Ca^2+^ channel *Fwe* (F) contain very small (0.1-1.5 µm) immature, intact secretory vesicles positive for both GFP and DsRed. Interestingly, these cells also include degradative (positive for DsRed only) large (4-8 µm) acidic glue granules (crinosomes) indicated by yellow arrowheads in the right insets. The boxed regions in panels A-F are shown enlarged on the right side of each panel. Green and magenta channels of merged images are also shown separately as indicated. Bars: 20 μm (A-F), 5 μm (A-F right insets)


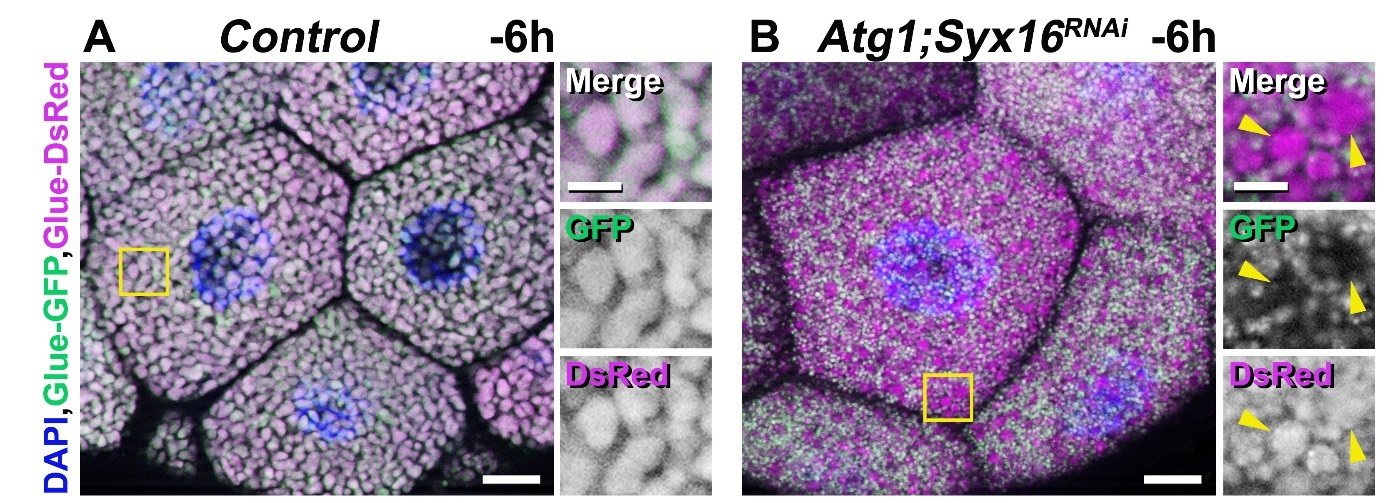


**Figure S2 Silencing of Atg1 and Syx16 genes simultaneously in salivary gland cells.**

(A-B) Degradation of small glue granules in the salivary gland cells of wandering L3 (-6h RPF) animals co-expressing Glue-GFP/Glue-DsRed (GlueFlux) reporters and the double RNAi-construct of Atg1 and Syx16 genes. (A) Control wandering L3 stage (-6h RPF) larval salivary gland cells normally contain large (3-3.5 µm), intact (GFP- and DsRed-double positive) glue granules. Compared to the control, salivary gland cells with Atg1-Syx16 double RNAi causes the accumulation of immature (1-1,5 µm), intact, glue granules and large (3-6 µm), acidic structures in these cells (indicated by yellow arrowheads in the right insets) which strongly suggests premature glue degradation at wandering (-6h RPF) stage. Bars: 20 μm (A-B), 5 μm (A-B right insets)
